# Effectiveness of sustained leisure-time physical activity strategies for obesity-related cancer prevention: an emulated target trial in a prospective US cohort

**DOI:** 10.1186/s12916-025-04417-z

**Published:** 2025-10-27

**Authors:** Valeria Elahy, Yu-Han Chiu, Alpa V. Patel, Erika Rees-Punia, Marjorie L. McCullough, Anita R. Peoples, Ying Wang

**Affiliations:** 1 Department of Population Science, American Cancer Society, Atlanta, GA 30303 USA; 2 Department of Epidemiology, Brown University School of Public Health, Providence, RI 02903 USA

**Keywords:** Physical activity, Cancer prevention, Obesity-related cancers, Target trial emulation, Parametric g-formula, Leisure-time physical activity, ACS guidelines, Causal inference, Older adults, Chronic disease prevention

## Abstract

**Background:**

Obesity-related cancers account for 40% of US cancer cases, and their global burden continues to rise. Cancer prevention guidelines recommend 150–300 min of moderate or 75–150 min of vigorous-intensity activity per week (7.5–15 MET-hrs/wk). However, the long-term causal effect of sustained leisure-time moderate-to-vigorous intensity physical activity (MVPA) on obesity-related cancer risk has not been quantified.

**Methods:**

We emulated a target trial using data from 60,958 cancer-free adults in the Cancer Prevention Study-II Nutrition Cohort (2001–2013) to estimate 11-year risks of obesity-related cancers under four sustained MVPA strategies: (1) no intervention (observed MVPA); (2) below recommendations (> 0– < 7.5 MET-hrs/wk); (3) meeting recommendations (7.5–15 MET-hrs/wk); and (4) exceeding recommendations (> 15 MET-hrs/wk). MVPA was self-reported every 2 years. The parametric g-formula was used to estimate cancer risk under each strategy among all eligible participants and stratified by pre-intervention MVPA (meeting vs. not meeting recommendations 2 years prior to intervention).

**Results:**

Over a median follow-up of 11.4 years (IQR 6.9–11.8), 4344 obesity-related cancers were diagnosed. Under no intervention, median baseline MVPA was 12.8 MET-hrs/wk (IQR 4.5–24.5) overall, 20.5 (IQR 15.2–30.8) among those meeting (*n* = 38,558), and 4.3 (IQR 1.5–6.2) among those not meeting recommendations pre-intervention (*n* = 22,400). The estimated 11-year cancer risk under no intervention was 8.2% overall, 8.1% among those meeting, and 8.7% among those not meeting recommendations pre-intervention. Compared to no intervention, risk differences were 0.18% (95% CI: 0.05% to 0.37%) for below-recommendation MVPA, 0.08% (95% CI: − 0.05% to 0.19%) for meeting, and − 0.18% (95% CI: − 0.44% to 0.01%) for exceeding recommendations. Among those meeting recommendations pre-intervention, risk differences were 0.34% (95% CI: 0.11% to 0.65%), 0.09% (95% CI: − 0.06% to 0.26%), and − 0.21% (95% CI: − 0.45% to − 0.05%), respectively. Among those not meeting recommendations, corresponding risk differences were − 0.02% (95% CI: − 0.31% to 0.27%), − 0.04% (95% CI: − 0.21% to 0.15%), and − 0.10% (95% CI: − 0.38% to 0.14%).

**Conclusions:**

We estimated that, compared to no intervention, sustaining MVPA volumes below recommendations may modestly increase obesity-related cancer risk over 11 years, whereas exceeding recommendations may modestly reduce risk, particularly among participants already meeting the recommendations prior to intervention.

**Supplementary Information:**

The online version contains supplementary material available at 10.1186/s12916-025-04417-z.

## Background

The 2018 American College of Sports Medicine Roundtable [1] concluded that there is strong evidence supporting the role of physical activity in the prevention of several types of cancer, including breast, colon, endometrial, kidney, bladder, esophageal, and stomach, as well as those mediated through obesity [2, 3]. Physical activity is believed to influence cancer risk through its effect on glucose metabolism, chronic inflammation, immune surveillance, hormonal regulation, and direct changes in the tumor microenvironment [4–7].

These biological pathways are particularly relevant to obesity-related cancers, where excess adiposity drives metabolic and hormonal dysregulation [3]. The International Agency for Research on Cancer (IARC) has identified 13 cancer types as obesity-related: adenocarcinoma of the esophagus, postmenopausal breast, colon, rectum, uterus, gallbladder, stomach, kidney, liver, ovary, pancreas, thyroid, meningioma, and multiple myeloma [3]. Together, these cancers account for approximately 40% of all cancer cases in the United States (US), contributing substantially to the national and global cancer burden [3, 8, 9].

To help mitigate cancer burden, the American Cancer Society (ACS) and World Cancer Research Fund (WCRF) recommend adults engage in 150–300 min of moderate-intensity or 75–150 min of vigorous-intensity activity per week (equivalent to 7.5–15 metabolic equivalent hours per week [MET-hrs/wk]) for cancer prevention [10, 11]. Meeting or exceeding these recommendations is considered optimal, yet nearly two-thirds of adults in the US and one-third of adults globally do not meet these guidelines [12, 13]. The challenge of achieving these recommendations becomes even more daunting in Western societies, largely due to increasingly sedentary lifestyles and changes in occupational and recreational patterns [14] as well as increasing aging population [15].

Despite the well-established biological plausibility, supporting guidelines and strong evidence linking higher volumes of leisure-time aerobic moderate-to-vigorous intensity physical activity (MVPA) to a lower risk of several obesity-related cancers [1, 16–19], to our knowledge, no study to date has quantified the effect of long-term *sustained* adherence to physical activity recommendations on the incidence of all obesity-related cancers combined.

Although a randomized controlled trial (RCT) would offer the strongest causal evidence, long-term lifestyle interventions are impractical due to high cost, adherence challenges, and required extended follow-up duration. In the absence of an RCT, emulating a target trial using high-quality observational data with clearly defined intervention strategies provides a practical alternative. Several studies have applied this approach to estimate the effects of adherence to lifestyle recommendations on cancer incidence [20] and survival [21].

In this study, we emulated a target trial using data from the prospective Cancer Prevention Study-II (CPS-II) Nutrition Cohort to estimate the 11-year risk of obesity-related cancers under sustained MVPA strategies aligned with ACS recommendations for cancer prevention [22, 23]. Outcomes included the incidence of all obesity-related cancers combined, as well as five site-specific obesity-related cancers with a sufficient number of cases for reliable risk estimation (colorectal, postmenopausal breast, endometrium, pancreas, and kidney cancers). We also conducted a secondary analysis stratified by pre-intervention MVPA to assess whether effects differed by prior adherence to activity recommendations.

## Methods

We structured this section in five parts. First, we describe the observational data source. Next, we specify the protocol for a hypothetical pragmatic trial (“target trial”) that would answer our causal question regarding sustained adherence to the physical activity recommendations and obesity-related cancer risk. We then describe how we emulated the target trial protocol using the observational data, including the modifications necessary to align the target trial protocol and the statistical methods used. Finally, we describe the sensitivity analyses conducted to assess the robustness of our findings.

### Data source

To emulate the target trial, we used data from the CPS-II Nutrition Cohort, a large prospective US cohort established in 1992/1993 as a subcohort of the CPS-II Cohort, originally launched in 1982 [24]. CPS-II Nutrition Cohort enrolled over 180,000 adult males and females from 21 states to investigate the relationship between a broad range of lifestyle factors, including diet, alcohol consumption, vitamin supplementation, tobacco use, physical activity, hormone and aspirin use, air pollution exposure, and family history of cancer and cancer outcomes [24, 25]. Data on education, race, and date of birth were collected in 1982, while medical history, physical activity, alcohol consumption, smoking history, diet, and other lifestyle factors were assessed through detailed questionnaires in the 1992/1993 survey [25, 26]. Beginning in 1997, biennial surveys updated covariate data and identified new self-reported cancer diagnoses, which were verified via medical records, linkage with state cancer registries, or the National Death Index [25, 27]. For this analysis, cancer incidence was defined as first verified primary cancer diagnosis or cancer as primary cause of death and was ascertained from the return of the 2001 CPS-II Nutrition Cohort follow-up survey (defined as baseline) through June 30, 2013 (administrative end of follow-up). Pre-baseline was defined as the return of the 1999 survey. Additional details on covariate derivation are provided in Additional file 1: Additional methods.

The CPS-II Nutrition Cohort assessed MVPA using a self-administered questionnaire adapted from the validated Nurses’ Health Study II physical activity and inactivity assessment tool, ensuring comparability and reliability [28]. We used MVPA data from the 1999, 2001, 2005, 2007, 2009, and 2011 CPS-II Nutrition Cohort surveys for this analysis [29]. Leisure-time MVPA was defined as voluntary activities aimed at maintaining fitness and health, with moderate intensity classified as ≥ 3 metabolic equivalents (MET) and vigorous intensity as ≥ 6 MET [30]. Participants reported average weekly time spent on leisure-time aerobic MVPA (e.g., walking, jogging, bicycling, swimming, tennis, aerobics, and dancing) [19, 29] and each activity was assigned a standard metabolic equivalent (MET) value based on intensity: walking (3.5 MET), biking (4.0 MET), jogging/running (7.0 MET), aerobics (4.5 MET), swimming (7.0 MET), dancing (3.5 MET), and tennis (6.0 MET) [19, 30]. Weekly MET-hours (MET-hrs/wk) of MVPA were then calculated by multiplying each activity’s MET value by the reported weekly duration.

### Target trial specifications

We conceptualized a target trial enrolling cancer-free adults aged ≥ 50 years with BMI ≥ 18.5 kg/m^2^ and no major cardiovascular events (heart attack, angina, coronary artery disease diagnosis, coronary bypass, angioplasty, stroke, and transient ischemic attack) within 2 years of baseline. Participants would be randomly assigned to one of four intervention strategies aligned with the 2018 US Physical Activity Guidelines, the WCRF, and the ACS recommendations for cancer prevention [10, 11, 13]:

1. No intervention (natural course of MVPA observed in the study sample, i.e., continuing one’s MVPA practices without intervention)
2. Below MVPA recommendations (> 0– < 7.5 MET-hrs/wk)
3. Meeting MVPA recommendations (7.5–15 MET-hrs/wk)
4. Exceeding MVPA recommendations (> 15 MET-hrs/wk)

Strategies 2–4 were designed as threshold strategies, requiring participants to maintain their assigned MVPA volume throughout the 11-year follow-up, with assessments of adherence via monthly survey questionnaires [31]. For example, those assigned to the 7.5–15 MET-hrs/wk strategy would be required to maintain MVPA within the assigned range. At the start of every follow-up period, they would be asked how much MVPA they would get if assigned to no intervention. If they intend to get MVPA volume below 7.5 MET-hrs/wk, they would be instructed to increase it exactly to 7.5 MET-hrs/wk, and if they intend to get MVPA volume above 15 MET-hrs/wk, they would be instructed to limit it exactly to 15 MET-hrs/wk. They would be instructed to make no change to their intended MVPA volume if it was within the assigned range. Participants would be excused from intervention upon developing a major cardiovascular event that could preclude engagement in leisure-time MVPA during follow-up.

The outcomes would be the 11-year risks of all 13 obesity-related cancers combined and individually, as defined by the IARC [3].

Follow-up would begin at baseline and continue until the first cancer diagnosis, loss to follow-up, unverified cancer diagnosis report, death, or the administrative end of follow-up, whichever occurs first. Participants would be censored at their last cancer-free follow-up survey if they missed a survey or had an unverified cancer report.

### Target trial emulation

**Fig. 1:**
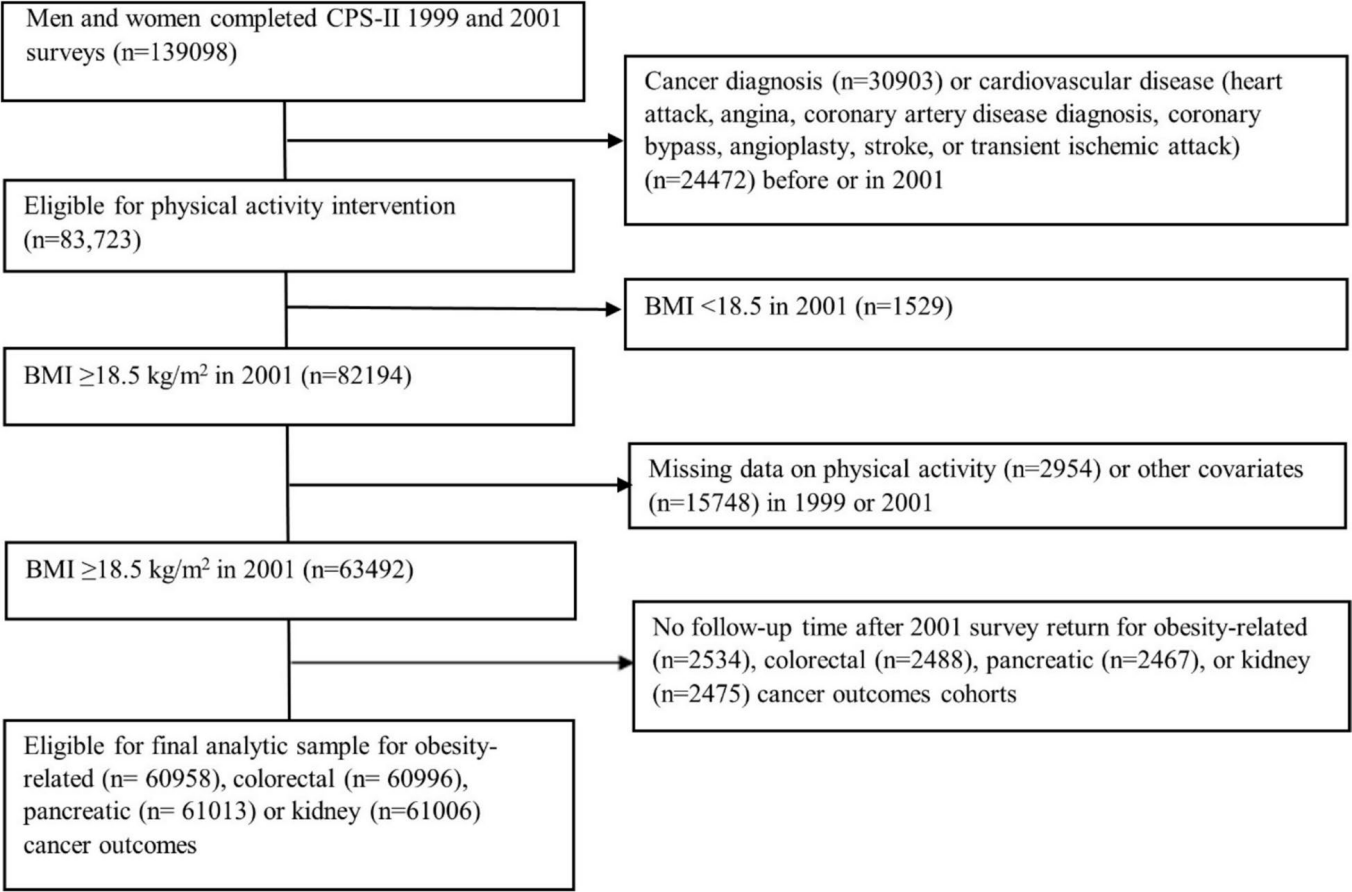
Flowchart of the eligible individuals for the emulated target trial of recreational physical activity and obesity-related, colorectal, pancreatic, or kidney cancer outcomes in the Cancer Prevention Study-II Cohort (2001–2013)

We emulated the specified trial using the data of CPS-II Nutrition Cohort participants who completed the 1999 and 2001 surveys, excluding those with missing MVPA or covariate data at baseline or pre-baseline (Table 1, Fig. 1, Additional file 2: Fig. S1). Each eligible participant was assigned to all four intervention strategies at baseline (2001). To emulate randomization, we adjusted for baseline (2001) and pre-baseline (1999) covariates known to influence physical activity and cancer risk (Additional file 3: Table S1), assuming exchangeability conditional on these covariates [32]. The causal contrast of interest was the observational analog of the per-protocol effect, defined as the effect had all participants adhered to the assigned strategy unless they developed a major cardiovascular event [33]. For primary outcomes, all 13 obesity-related cancers were included in the definition of the combined obesity-related cancers. For secondary outcomes, we considered all 13 site-specific obesity-related cancers, but limited analyses to five cancer types with sufficient case counts for reliable risk estimation (colorectal, postmenopausal breast, endometrial, pancreatic, and kidney cancers).

**Table 1:** Emulation of a target trial of leisure-time aerobic moderate-to-vigorous intensity physical activity interventions using observational data from the Cancer Prevention Study-II Nutrition Cohort (2001–2013)

Component	Target trial specifications	Target trial emulation
Aim	Estimate the effect of sustained leisure-time aerobic moderate-to-vigorous intensity physical activity (MVPA) strategies on 11-year obesity-related cancer risk	Same
Baseline definition	2001	Same. Baseline is defined as the return date of the 2001 CPS-II Nutrition Cohort survey to allow adjustment for pre-baseline covariates from 1999
Eligibility criteria	• Age ≥ 50 years • No prevalent cancer • BMI ≥ 18.5 kg/m^2^ • No cardiovascular events (heart attack, angina, coronary artery disease diagnosis, coronary bypass, angioplasty, stroke, or TIA) in the past 2 years	Same. Additional exclusions: • Non-response to 1999 or 2001 surveys • Missing data on MVPA or other covariates in 1999 or 2001
Treatment strategies	1) No intervention (natural course of MVPA) 2) Below recommended MVPA (> 0 to < 7.5 MET-hrs/wk) 3) Meeting recommended MVPA (7.5 to 15 MET-hrs/wk) 4) Exceeding recommended MVPA (> 15 MET-hrs/wk) Participants are expected to sustain assigned MVPA volume until the end of the follow-up. Individuals are excused from adhering to the strategies 2–3 if they report major cardiovascular	Same
Treatment assignment	Random assignment at baseline, with strategy awareness. Each participant is assigned to one strategy. Monthly assessments of adherence	Each participant is assigned to all four strategies. To emulate randomization, we adjusted for baseline (age, family history of cancer, sex, race, education, BMI, diabetes, smoking history) and pre-baseline covariates (physical activity, diet quality (assessed using ACS diet score), alcohol consumption). Adherence was assessed biennially, consistent with CPS-II survey cycles, assuming participants sustained their MVPA volume over the 2-year intervals
Follow-up	From baseline until loss to follow-up, cancer diagnosis, death, or administrative end of follow-up (June 30, 2013), whichever occurs first	Same. Loss to follow-up is defined as a survey non-response; participants reporting unverified cancer were censored at the last cancer-free survey
Outcome	11-year risk of IARC-defined obesity-related cancers combined (adenocarcinoma of the esophagus, postmenopausal breast, colon, rectum, uterus, gallbladder, gastric cardia (upper stomach), kidney, liver, ovary, pancreas, thyroid, meningioma, and multiple myeloma) and by site Cancer cases are verified through medical records, registries, or the National Death Index. Competing events are defined as death due to non-obesity-related cancer or due to non-cancer cause	Same. For site-specific analyses, analyses were limited to colorectal, postmenopausal breast, endometrial, pancreatic, and kidney cancers due to power considerations
Causal contrast of interest	Intention-to-treat and per-protocol effect Estimation of the total effect of intervention strategies on cancer risk	Observational analog of the per-protocol effect
Statistical analyses	Intention to treat analysis Per-protocol analysis: using the parametric g-formula to estimate 11-year cancer risk under each MVPA strategy versus no intervention, adjusting for pre-baseline, baseline, and time-varying factors related to adherence and loss to follow-up	Same as per-protocol analysis

### Statistical analysis

We used the parametric g-formula to estimate the 11-year risk of cancer under sustained adherence to the intervention strategies, adjusting for time-varying confounders affected by prior exposure [34–36]. The estimated risk under each strategy is a weighted average of the individual risks conditional on each participant’s time-varying covariate and treatment (MVPA) history, with the distribution of covariate histories under that strategy serving as weights (Table S1) [34, 37]. The process involved four steps: (1) We modeled the conditional distribution of outcomes and covariates at each follow-up period, given covariate and physical activity history. (2) Using Monte Carlo simulation, we generated time-varying covariate histories consistent with each hypothetical intervention strategy. (3) Based on predicted hazards from the models above, we calculated the outcome probability under each hypothetical strategy for each individual. (4) The population-level risk (i.e., cumulative incidence) under each strategy was then estimated by averaging the individual-specific predicted risk.

Models included baseline (age, sex, race, education, family history of cancer, smoking history, BMI, diabetes, CVD), pre-baseline (MVPA, diet quality, and alcohol consumption), and time-varying covariates (BMI, MVPA, alcohol consumption, diabetes, CVD). MVPA was truncated at the 99th percentile to minimize the influence of outliers. Missing follow-up covariate data were carried forward from prior values, with time since the last update included in the model.

We calculated percentile-based 95% confidence intervals for all estimates using nonparametric bootstrapping. For each strategy, we also calculated the proportion of participants who would have to be “intervened” on to maintain adherence over an 11-year period [35].

We compared the estimated 11-year risk under each intervention strategy with that of a reference group using risk ratios (RR) and absolute risk differences (RD). For the primary analysis, the reference group was the “no intervention” strategy, representing the natural course of MVPA exposure in the absence of an intervention on physical activity, while intervening to eliminate loss to follow-up [38, 39]. In secondary analyses, we used the “not meeting recommended MVPA” strategy as the reference group to explore the potential benefits of meeting or exceeding MVPA recommendations compared to not meeting them.

All estimates represent the total effect estimands, accounting for all causal pathways between the interventions and outcomes, including those potentially mediated by competing events (i.e., death due to non-cancer causes or cancer not of interest) [40, 41]. Additional file 2: Fig. S2 illustrates an assumed relationship between the treatment, covariates, and the outcomes at two follow-up time points under which the competing event may mediate the treatment’s effect on the outcome.

To explore heterogeneity, we stratified the analyses by pre-baseline MVPA (< 7.5 MET-hrs/wk and ≥ 7.5 MET-hrs/wk) to separately assess the effect among participants who had met and had not met the MVPA recommendation before the intervention [42]. We also stratified analyses by sex and by baseline BMI (18.5– < 25 kg/m^2^ and ≥ 25 kg/m^2^) [43].

### Sensitivity analyses

We conducted several sensitivity analyses to assess the robustness of our findings by (1) lagged covariate data by 2 years to ensure they preceded physical activity data (estimating 9-year risk instead of 11-year risk) [44]; (2) using different functional forms BMI (linear and log-transformed); (3) altering the temporal ordering of time-varying covariates reported in the same questionnaire when modeling their joint distribution; (4) restricting baseline age to < 90 years and excusing participants from adherence requirements after age 90 due to limited data availability in this age group [25]; (5) excluding participants with baseline chronic obstructive pulmonary disease (COPD) and excusing from the adherence if participants developed COPD during the follow-up; (6) including participants with history of cardiovascular events at baseline and not excusing them from adherence after subsequent events; (7) adjusted for baseline and time-varying cancer screening history (breast, colorectal, prostate) to address the potential confounding effect of healthcare-seeking behavior; and finally (8) excluding current and recent smokers (< 20 years before baseline) [19, 45].

To further evaluate the validity of our modeling approach, we used cardiovascular disease (CVD) mortality (ICD-10 codes I00–I99) as a positive control outcome, where strong effects of MVPA are expected [19, 46]. For breast and endometrial cancer analyses, we adjusted for parity and age at first birth to account for reproductive factors [47, 48].

Analyses were completed using the Science Cloud analytic platform (SAS) developed by Manifold, Inc (Additional file 1: Sample program).

## Results

### Study population

The emulated target trial included 60,958 participants from the CPS-II Nutrition Cohort. At baseline (2001), the median age was 70.0 years (IQR 66.0–74.0), 97.8% were White, 62.5% were women, and 42.7% had a college degree or higher (Table 2). At baseline, 34.5% of participants reported MVPA of > 0– < 7.5 MET-hrs/wk, 23.6% reported 7.5–15 MET-hrs/wk, and 35.9% reported > 15 MET-hrs/wk. During follow-up, 5695 participants consistently reported MVPA volume within the same range, with 1237 (21.7%) consistently reporting > 0– < 7.5 MET-hrs/wk, 574 (10.1%) reporting 7.5–15 MET-hrs/wk, and 3546 (62.3%) reporting > 15 MET-hrs/wk.

**Table 2:** Baseline^a^ characteristics of 60,958 participants eligible for emulated target trial of leisure-time aerobic moderate-to-vigorous intensity physical activity (MVPA) interventions and obesity-related cancer risk in the Cancer Prevention Study-II Nutrition Cohort (2001–2013)

**Characteristic**		***N* (%) or median (IQR)**
**Age, years, median (IQR)**		70 (66–74)
**Sex**^**f**^**, *n* (%)**	Female	38,102 (62.5)
	Male	22,856 (37.5)
**Race/ethnicity**^**f**^**, *n* (%)**	White/White Hispanic	59,645 (97.8)
	Black/Black Hispanic	609 (1.0)
	Other/unknown	704 (1.2)
**Education**^**f**^**, *n* (%)**	High school graduate or less	17,719 (29.1)
	Some college	17,237 (28.3)
	College graduate or unknown	26,002 (42.7)
**Smoking history, *n* (%)**	Non-smoker	30,248 (50.0)
	Quit ≥ 30 y ago	15,355 (25.4)
	Quit 20– < 30 y ago	5820 (9.6)
	Quit 10– < 20 y ago	4745 (7.8)
	Quit < 10 y ago	2110 (3.5)
	Current smoker	2181 (3.6)
**BMI, *n* (%)**	18.5– < 25 kg/m^2^	26,858 (44.1)
	25– < 30 kg/m^2^	23,929 (39.3)
	≥ 30 kg/m^2^	10,171 (16.7)
**Family history of cancer**^**b**^**, *n* (%)**		33,845 (55.5)
**MVPA, *n* (%)**	0 MET-hrs/wk	3669 (6.0)
	> 0– < 7.5 MET-hrs/wk	21,019 (34.5)
	7.5–15 MET-hrs/wk	14,411 (23.6)
	> 15 MET-hrs/wk	21,859 (35.9)
**COPD, *n* (%)**		1914 (3.1)
**Diabetes**^**c**^**, *n* (%)**		6991 (11.5)
**Pre-/peri-menopausal status**^**g**^^**,i**^**, *n* (%)**		167 (0.4)
**Mammogram screening**^**g**^**, *n* (%)**	Never	828 (2.2)
	Recent	34,774 (91.4)
	Not recent	2433 (6.4)
**Endoscopy**^**d**^** screening, *n* (%)**	Never	15,472 (26.7)
	Ever	42,442 (73.3)
**PSA screening**^**h**^**, *n* (%)**	Never	1210 (5.4)
	Recent	19,564 (86.8)
	Not recent	1761 (7.8)
**Parity**^**g**^**, *n* (%)**	Nulliparous	2787 (7.3)
	1–2 births	12,676 (33.3)
	3 or more births	21,976 (57.7)
	Unknown	663 (1.7)
**Age at first birth**^**g**^**, *n* (%)**	Nulliparous	2787 (7.3)
	< 20 years	3323 (8.7)
	20–24 years	18,214 (47.8)
	25–29 years	10,271 (27.0)
	30 or more years	2757 (7.2)
	Unknown	750 (2.0)
**ACS diet score**^**e,i**^**, median (IQR)**		6.3 (4.8–7.8)
**Alcohol, servings/day**^**i**^**, median (IQR)**		0.1 (0–0.9)

Abbreviations: *ACS*, American Cancer Society; *BMI*, body mass index; *COPD*, chronic obstructive pulmonary disease; *MET*, metabolic equivalent of task; *MVPA*, moderate-to-vigorous intensity physical activity; *PSA*, prostate-specific antigen

^a^Baseline refers to the return date of the 2001 survey. Some characteristics are assessed in earlier survey cycles if specified

^b^Family history (among parents, siblings, and children) of colorectal, breast, prostate, pancreatic, and ovarian cancer was self-reported in 2001

^c^Diabetes diagnosis and insulin use

^d^Sigmoidoscopy and colonoscopy

^e^The ACS diet score (out of 12 points) is used to quantify adherence to the food-based recommendations of the ACS Nutrition and Physical Activity Guidelines for Cancer Prevention

^f^Assessed in 1982

^g^Assessed only in females ([%] out of females)

^h^Assessed only in males ([%] out of males)

^i^Assessed in 1999

During the 11-year follow-up (median, 11.4 years; IQR 6.9–11.8), 4344 obesity-related cancers were identified, including 876 colorectal, 1936 breast, 295 endometrial, 320 pancreatic, and 236 kidney cancers (Additional file 3: Table S2).

### Estimated 11-year cancer risk

Under no intervention, the estimated 11-year risk was 8.2% for all obesity-related cancers combined, 1.7% for colorectal cancer, 5.8% for female postmenopausal breast cancer, 0.9% for endometrial cancer, 0.6% for pancreatic cancer, and 0.5% for kidney cancer (Table 3). Compared with no intervention, the estimated risk under the > 0– < 7.5 MET-hrs/wk strategy was higher by 0.18% (95% CI, 0.05% to 0.37%) for all obesity-related cancers combined, 0.10% (95% CI, 0.02% to 0.19%) for colorectal cancer, and 0.14% (95% CI, 0.04% to 0.28%) for breast cancer. Estimated RD for endometrial cancer (− 0.03%; 95% CI, − 0.12% to 0.00%), pancreatic cancer (− 0.02%; 95% CI, − 0.06% to 0.03%), and kidney cancer (0.00%; 95% CI, − 0.04% to 0.04%) were close to null. RD for 7.5–15 MET-hrs/wk and > 15 MET-hrs/wk strategies were close to null across all outcomes, except for colorectal cancer under the > 15 MET-hrs/wk strategy (RD: − 0.16%; 95% CI, − 0.31% to − 0.04%). Across all outcomes, the average proportion of participants required to change their behavior to achieve adherence to every strategy at every assessment point ranged from 37.0% to 45.0%. Minor differences between confidence intervals for RR and RD measures (e.g., RR including 1.00 while RD does not cross 0.00) reflect rounding, not estimation inconsistencies. Additional file 2: Fig. S3 provides additional details on survival curves for intervention strategies.

**Table 3:** Estimated 11-year risks^a^ of all obesity-related cancers, female postmenopausal breast cancer, colorectal cancer, pancreatic cancer, endometrial cancer, and kidney cancer under leisure-time aerobic moderate-to-vigorous intensity physical activity (MVPA) strategies compared to physical activity under no intervention in the Cancer Prevention Study-II Nutrition Cohort (2001–2013)

Intervention	Estimated risk (95% CI), %	Risk ratio (95% CI), %	Risk difference (95% CI)	Average % intervened on	Cumulative % intervened on^d^
**Obesity-related cancer**					
No intervention^b,c^	8.2 (7.9, 8.4)	1.00 (reference)	0 (reference)	0.0	0.0
> 0– < 7.5 MET-hrs/wk	8.4 (8.1, 8.7)	1.02 (1.01, 1.04)	0.18 (0.05, 0.37)	37.1	93.7
7.5–15 MET-hrs/wk	8.3 (8.0, 8.5)	1.01 (0.99, 1.02)	0.08 (− 0.05, 0.19)	43.8	97.2
> 15 MET-hrs/wk	8.0 (7.6, 8.3)	0.98 (0.95, 1.00)	− 0.18 (− 0.44, 0.01)	37.2	90.6
**Colorectal cancer**					
No intervention^b,c^	1.7 (1.6, 1.8)	1.00 (reference)	0 (reference)	0.0	0.0
> 0– < 7.5 MET-hrs/wk	1.8 (1.7, 2.0)	1.06 (1.01, 1.11)	0.10 (0.02, 0.19)	37.2	93.7
7.5–15 MET-hrs/wk	1.7 (1.6, 1.8)	0.98 (0.92, 1.03)	− 0.03 (− 0.13, 0.06)	43.7	97.1
> 15 MET-hrs/wk	1.6 (1.4, 1.7)	0.91 (0.83, 0.98)	− 0.16 (− 0.31, − 0.04)	37.1	90.4
**Breast cancer**					
No intervention^b,c^	5.8 (5.5, 5.9)	1.00 (reference)	0 (reference)	0.0	0.0
> 0– < 7.5 MET-hrs/wk	5.9 (5.6, 6.1)	1.02 (1.01, 1.05)	0.14 (0.04, 0.28)	37.5	93.8
7.5–15 MET-hrs/wk	5.9 (5.5, 6.1)	1.01 (0.97, 1.04)	0.08 (− 0.15, 0.22)	45.0	97.6
> 15 MET-hrs/wk	5.7 (5.3, 6.0)	0.98 (0.93, 1.02)	− 0.12 (− 0.39, 0.12)	39.8	93.4
**Endometrial cancer**					
No intervention^b,c^	0.9 (0.8, 1.0)	1.00 (reference)	0 (reference)	0.0	0.0
> 0– < 7.5 MET-hrs/wk	0.9 (0.8, 1.0)	0.97 (0.86, 1.01)	− 0.03 (− 0.12, 0.00)	37.4	94.0
7.5–15 MET-hrs/wk	0.9 (0.8, 1.0)	1.04 (0.94, 1.10)	0.03 (− 0.05, 0.09)	44.9	97.5
> 15 MET-hrs/wk	1.0 (0.8, 1.2)	1.08 (0.99, 1.29)	0.07 (− 0.01, 0.28)	39.9	93.4
**Pancreatic cancer**					
No intervention^b,c^	0.6 (0.6, 0.7)	1.00 (reference)	0 (reference)	0.0	0.0
> 0– < 7.5 MET-hrs/wk	0.6 (0.5, 0.7)	0.97 (0.90, 1.05)	− 0.02 (− 0.06, 0.03)	37.0	93.6
7.5–15 MET-hrs/wk	0.7 (0.6, 0.7)	1.04 (0.99, 1.11)	0.02 (− 0.01, 0.07)	43.6	97.1
> 15 MET-hrs/wk	0.7 (0.6, 0.8)	1.08 (0.97, 1.21)	0.05 (− 0.02, 0.13)	37.1	90.6
**Kidney cancer**					
No intervention^b,c^	0.5 (0.4, 0.5)	1.00 (reference)	0 (reference)	0.0	0.0
> 0– < 7.5 MET-hrs/wk	0.5 (0.4, 0.5)	1.01 (0.92, 1.08)	0.00 (− 0.04, 0.04)	37.1	93.7
7.5–15 MET-hrs/wk	0.5 (0.4, 0.5)	1.05 (0.97, 1.11)	0.02 (− 0.01, 0.05)	43.7	97.2
> 15 MET-hrs/wk	0.5 (0.4, 0.6)	1.03 (0.88, 1.19)	0.02 (− 0.05, 0.09)	37.2	90.6

Abbreviations: *CI*, confidence interval; *MET*, metabolic equivalent of task; *MVPA*, moderate-to-vigorous intensity physical activity

^a^Estimates are based on the parametric g-formula, adjusting for baseline (age, family history of cancer, sex, race, education, BMI, diabetes, smoking history) and pre-baseline (physical activity, diet quality, alcohol consumption) and time-varying covariates (BMI, physical activity, alcohol consumption, diabetes, CVD). Individuals were not censored upon the development of a competing event to estimate the total effect of the intervention. A nonparametric bootstrapping was used to calculate 95% confidence intervals for all estimates

^b^No intervention or “natural course” refers to observing and analyzing the effect of natural physical activity in the eligible population without any intervention

^c^Among 60,958 participants, including 38,131 females, there were 4344 obesity-related cancers, including 876 colorectal cancers, 1936 postmenopausal breast cancers, 295 endometrial cancers, 320 pancreatic cancers, and 236 kidney cancers. The inverse probability weighted risk under no intervention was 8.3%, 1.7%, 5.9%, 0.9%, 0.7%, and 0.5% for all obesity-related, colorectal, postmenopausal breast, endometrial, pancreatic, and kidney cancers, respectively

^d^Cumulative proportion of eligible participants who would have to change their physical activity level at any follow-up period to keep adhering to the specific intervention strategy

When using the “not meeting recommended MVPA” strategy as the reference group, estimated 11-year risks were modestly lower for all obesity-related cancers under both the 7.5–15 MET-hrs/wk strategy (− 0.11%; 95% CI, − 0.34% to 0.05%) and the > 15 MET-hrs/wk strategy (− 0.36%; 95% CI, − 0.79% to − 0.05%). Similarly, for colorectal cancer, estimated risks were also lower under both the 7.5–15 MET-hrs/wk strategy (− 0.13%; 95% CI, − 0.29% to − 0.01%) and the > 15 MET-hrs/wk strategy (− 0.26%; 95% CI, − 0.50% to − 0.07%). No clear differences were observed for breast, endometrial, pancreatic, or kidney cancer (Additional file 3: Table S3).

### Stratified analyses by pre-baseline MVPA

Pre-intervention, 22,400 (36.7%) participants reported not meeting MVPA recommendations, and 38,558 (63.3%) participants reported meeting or exceeding them. Under the no intervention strategy, the estimated 11-year risks among participants not meeting and meeting recommendations were 8.5% and 8.0% for all obesity-related cancers, 1.7% and 1.7% for colorectal cancer, 5.8% and 5.8% for female postmenopausal breast cancer, 1.0% and 0.9% for endometrial cancer, 0.6% and 0.7% for pancreatic cancer, and 0.5% and 0.5% for kidney cancer, respectively. Compared to no intervention, the estimated risks under the > 0– < 7.5 MET-hrs/wk strategy were higher for all obesity-related cancers (0.34%; 95% CI, 0.11% to 0.65%) and for colorectal cancer (0.18%; 95% CI, 0.07% to 0.25%) among participants meeting recommendations pre-intervention, while the effect was null among those not meeting recommendations pre-intervention. Compared to no intervention, the estimated risks under the > 15 MET-hrs/wk strategy were lower for all obesity-related cancers (− 0.21%; 95% CI, − 0.45% to − 0.05%) and for colorectal cancer (− 0.17%; 95% CI, − 0.25% to − 0.07%) among participants meeting recommendations pre-baseline and null among those not meeting recommendations pre-baseline (Table 4). When using the “not meeting recommended MVPA” strategy as the reference group, the estimated RD were similar but slightly more pronounced (Additional file 3: Table S4). RD for other site-specific cancers were generally close to null in both strata.

**Table 4:** Stratified analysis by pre-baseline (1999) MVPA. Estimated^a^ 11-year risk differences for all obesity-related cancers, female postmenopausal breast cancer, colorectal cancer, pancreatic cancer, endometrial cancer, and kidney cancer under leisure-time aerobic moderate-to-vigorous intensity physical activity (MVPA) compared to no intervention^b^ in the Cancer Prevention Study-II Nutrition Cohort (2001–2013)

Treatment strategy	Estimated 11-year risk difference (95% CI), %	
	**Pre-baseline MVPA < 7.5 MET-hrs/wk**	**Pre-baseline MVPA ≥ 7.5 MET-hrs/wk**
**Obesity-related cancer**^**c**^		
> 0– < 7.5 MET-hrs/wk vs. no intervention	0.02 (− 0.09, 0.14)	0.34 (0.11, 0.65)
7.5–15 MET-hrs/wk vs. no intervention	0.01 (− 0.30, 0.49)	0.14 (− 0.02, 0.31)
> 15 MET-hrs/wk vs. no intervention	− 0.06 (− 0.62, 0.48)	− 0.21 (− 0.46, − 0.05)
**Colorectal cancer**^**c**^		
> 0– < 7.5 MET-hrs/wk vs. no intervention	0.02 (− 0.04, 0.06)	0.18 (0.07, 0.25)
7.5–15 MET-hrs/wk vs. no intervention	− 0.05 (− 0.25, 0.09)	− 0.01 (− 0.10, 0.07)
> 15 MET-hrs/wk vs. no intervention	− 0.11 (− 0.37, 0.15)	− 0.17 (− 0.25, − 0.07)
**Breast cancer**^**c**^		
> 0– < 7.5 MET-hrs/wk vs. no intervention	0.05 (− 0.08, 0.22)	0.28 (− 0.06, 0.60)
7.5–15 MET-hrs/wk vs. no intervention	0.23 (− 0.15, 0.57)	0.12 (− 0.03, 0.28)
> 15 MET-hrs/wk vs. no intervention	0.06 (− 0.46, 0.52)	− 0.17 (− 0.39, 0.22)
**Endometrial cancer**^**c**^		
> 0– < 7.5 MET-hrs/wk vs. no intervention	0.02 (− 0.03, 0.10)	− 0.05 (− 0.16, 0.04)
7.5–15 MET-hrs/wk vs. no intervention	0.12 (− 0.09, 0.40)	0.00 (− 0.05, 0.07)
> 15 MET-hrs/wk vs. no intervention	0.04 (− 0.26, 0.45)	0.05 (− 0.04, 0.16)
**Pancreatic cancer**^**c**^		
> 0– < 7.5 MET-hrs/wk vs. no intervention	0.00 (− 0.06, 0.05)	− 0.06 (− 0.14, 0.01)
7.5–15 MET-hrs/wk vs. no intervention	− 0.04 (− 0.14, 0.11)	0.03 (− 0.01, 0.07)
> 15 MET-hrs/wk vs. no intervention	− 0.05 (− 0.24, 0.11)	0.07 (0.01, 0.13)
**Kidney cancer**^**c**^		
> 0– < 7.5 MET-hrs/wk vs. no intervention	− 0.02 (− 0.05, 0.01)	0.02 (− 0.03, 0.10)
7.5–15 MET-hrs/wk vs. no intervention	− 0.03 (− 0.10, 0.07)	0.03 (− 0.01, 0.06)
> 15 MET-hrs/wk vs. no intervention	0.02 (− 0.10, 0.16)	0.00 (− 0.06, 0.06)

Abbreviations: *CI*, confidence interval; *MET*, metabolic equivalent of task; *MVPA*, moderate-to-vigorous intensity physical activity

^a^Estimates are based on the parametric g-formula adjusting for baseline (age, family history of cancer, sex, race, education, BMI, diabetes, smoking history) and pre-baseline (physical activity, diet quality, alcohol consumption) and time-varying covariates (BMI, physical activity, alcohol consumption, diabetes, CVD). Individuals were not censored upon the development of a competing event to estimate the total effect of the intervention. A nonparametric bootstrapping was used to calculate 95% confidence intervals for all estimates

^b^No intervention or “natural course” refers to observing and analyzing the effect of natural physical activity in the eligible population without any intervention

^c^Observed 11-year inverse probability weighted risks under no intervention and sample sizes were as follows: for obesity-related cancer, 8.7% in pre-baseline MVPA < 7.5 MET-hrs/wk group (*N* = 22,400) and 8.1% pre-baseline MVPA ≥ 7.5 MET-hrs/wk group (*N* = 38,558); for colorectal cancer, 1.7% 8.1% in pre-baseline MVPA < 7.5 MET-hrs/wk group (*N* = 22,412) and 1.7% pre-baseline MVPA ≥ 7.5 MET-hrs/wk group (*N* = 38,584); for breast cancer among postmenopausal women, 5.9% in pre-baseline MVPA < 7.5 MET-hrs/wk group (*N* = 15,047) and 5.8% pre-baseline MVPA ≥ 7.5 MET-hrs/wk group (*N* = 23,084); for endometrial cancer, 1.0% in pre-baseline MVPA < 7.5 MET-hrs/wk group (*N* = 15,044) and 0.9% pre-baseline MVPA ≥ 7.5 MET-hrs/wk group (*N* = 23,082); for pancreatic cancer, 0.6% in pre-baseline MVPA < 7.5 MET-hrs/wk group (*N* = 22,414) and 0.7% pre-baseline MVPA ≥ 7.5 MET-hrs/wk group (*N* = 38,599); and for kidney cancer, 0.5% in pre-baseline MVPA < 7.5 MET-hrs/wk group (*N* = 22,412) and 0.4% pre-baseline MVPA ≥ 7.5 MET-hrs/wk group (*N* = 38,594)

### Stratified analyses by baseline BMI

At baseline, 26,858 participants (44.0%) had normal body weight, and 34,100 participants (56.0%) had overweight or obesity. Additional file 2: Fig. S4 provides additional details on mean BMI throughout the follow-up. Under the no intervention strategy, the estimated 11-year risks among participants with normal body weight and with overweight/obesity were 8.0% and 8.3% for all obesity-related cancers, 1.7% and 1.8% for colorectal cancer, 5.5% and 6.1% for female postmenopausal breast cancer, 0.6% and 1.1% for endometrial cancer, 0.6% and 0.7% for pancreatic cancer, and 0.3% and 0.6% for kidney cancer, respectively. Compared to no intervention, the estimated RDs for site-specific cancers were consistent across BMI strata and null, with some variability observed for all obesity-related cancers combined, colorectal cancer, and breast cancer (Table 5). Compared to no intervention, the risk under the > 0– < 7.5 MET-hrs/wk strategy was higher for all obesity-related cancers (0.22%; 95% CI, 0.05% to 0.42%) and for breast cancer (0.24%; 95% CI, 0.07% to 0.40%) among participants with overweight or obesity, while the effect was null among those with normal body weight. Compared to no intervention, the risk under the > 15 MET-hrs/wk strategy was lower for colorectal cancer (0.26%; 95% CI, − 0.43% to − 0.08%) among participants with normal body weight and null among those with overweight or obesity. Estimates for endometrial, pancreatic, and kidney cancers in the normal body weight group were not presented due to positivity violations and unstable estimates in those strata. When using the “not meeting recommended MVPA” strategy as the reference group, the estimated RD were similar but slightly more pronounced (Additional file 3: Table S5).

**Table 5:** Stratified analysis by baseline (2001) BMI. Estimated^a^ 11-year risk differences for all obesity-related cancers, female postmenopausal breast cancer, colorectal cancer, pancreatic cancer, endometrial cancer, and kidney cancer under leisure-time aerobic moderate-to-vigorous intensity physical activity (MVPA) strategies compared to no intervention^b^ in the Cancer Prevention Study-II Nutrition Cohort (2001–2013)

Treatment strategy	Estimated 11-year risk difference (95% CI), %	
	**Baseline BMI 18.5– < 25 kg/m**^**2**^	**Baseline BMI ≥ 25 kg/m**^**2**^
**Obesity-related cancer**^**c**^		
> 0– < 7.5 MET-hrs/wk vs. no intervention	0.11 (− 0.20, 0.36)	0.22 (0.05, 0.42)
7.5–15 MET-hrs/wk vs. no intervention	0.03 (− 0.14, 0.22)	0.11 (− 0.12, 0.29)
> 15 MET-hrs/wk vs. no intervention	− 0.10 (− 0.37, 0.23)	− 0.21 (− 0.56, 0.19)
**Colorectal cancer**^**c**^		
> 0– < 7.5 MET-hrs/wk vs. no intervention	0.15 (− 0.01, 0.26)	0.07 (− 0.01, 0.16)
7.5–15 MET-hrs/wk vs. no intervention	− 0.12 (− 0.22, 0.00)	0.06 (− 0.04, 0.16)
> 15 MET-hrs/wk vs. no intervention	− 0.26 (− 0.43, − 0.08)	− 0.05 (− 0.16, 0.12)
**Breast cancer**^**c**^		
> 0– < 7.5 MET-hrs/wk vs. no intervention	− 0.02 (− 0.23, 0.27)	0.24 (0.07, 0.40)
7.5–15 MET-hrs/wk vs. no intervention	0.12 (− 0.03, 0.31)	− 0.02 (− 0.22, 0.34)
> 15 MET-hrs/wk vs. no intervention	0.12 (− 0.19, 0.39)	− 0.37 (− 0.71, 0.15)
**Endometrial cancer**^**c,d**^		
> 0– < 7.5 MET-hrs/wk vs. no intervention	–	− 0.04 (− 0.11, 0.04)
7.5–15 MET-hrs/wk vs. no intervention	–	0.00 (− 0.12, 0.20)
> 15 MET-hrs/wk vs. no intervention	–	0.07 (− 0.14, 0.39)
**Pancreatic cancer**^**c,d**^		
> 0– < 7.5 MET-hrs/wk vs. no intervention	–	− 0.02 (− 0.09, 0.04)
7.5–15 MET-hrs/wk vs. no intervention	–	0.06 (0.01, 0.14)
> 15 MET-hrs/wk vs. no intervention	–	0.09 (− 0.01, 0.20)
**Kidney cancer**^**c,d**^		
> 0– < 7.5 MET-hrs/wk vs. no intervention	–	0.02 (− 0.03, 0.06)
7.5–15 MET-hrs/wk vs. no intervention	–	0.04 (− 0.02, 0.09)
> 15 MET-hrs/wk vs. no intervention	–	0.01 (− 0.08, 0.11)

Abbreviations: *CI*, confidence interval; *MET*, metabolic equivalent of task; *MVPA*, moderate-to-vigorous intensity physical activity

^a^Estimates are based on the parametric g-formula adjusting for baseline (age, family history of cancer, sex, race, education, BMI, diabetes, smoking history) and pre-baseline (physical activity, diet quality, alcohol consumption) and time-varying covariates (BMI, physical activity, alcohol consumption, diabetes, CVD). Individuals were not censored upon the development of a competing event to estimate the total effect of the intervention. A nonparametric bootstrapping was used to calculate 95% confidence intervals for all estimates

^b^No intervention or “natural course” refers to observing and analyzing the effect of natural physical activity in the eligible population without any intervention

^c^Observed 11-year inverse probability weighted risks under no intervention and sample sizes were as follows: for obesity-related cancer, 8.1% in BMI 18.5– < 25 kg/m^2^ (*N* = 26,858) and 8.5% in BMI ≥ 25 kg/m^2^ (*N* = 34,100); for colorectal cancer, 1.7% in BMI 18.5– < 25 kg/m^2^ (*N* = 26,876) and 1.7% in BMI ≥ 25 kg/m^2^ (*N* = 34,120); for breast cancer among postmenopausal women, 5.5% in BMI 18.5– < 25 kg/m^2^ (*N* = 18,778) and 6.2% in BMI ≥ 25 kg/m^2^ (*N* = 19,353); for endometrial cancer, 0.7% in BMI 18.5– < 25 kg/m^2^ (*N* = 18,776) and 1.2% in BMI ≥ 25 kg/m^2^ (*N* = 19,350); for pancreatic cancer, 0.6% in BMI 18.5– < 25 kg/m^2^ (*N* = 26,883) and 0.7% in BMI ≥ 25 kg/m^2^ (*N* = 34,130); and for kidney cancer, 0.3% in BMI 18.5– < 25 kg/m^2^ (*N* = 26,879) and 0.6% in BMI ≥ 25 kg/m^2^ (*N* = 34,127)

^d^Estimates for endometrial, pancreatic, and kidney cancer among participants with BMI 18.5– < 25 kg/m^2^ are not presented due to quasi-complete separation in the data, which suggests a violation of the positivity assumption. In these strata, sparse data limited the ability to support the modeled interventions without strong extrapolation, resulting in unstable and potentially unreliable estimates

### Stratified analyses by sex

Compared to no intervention, the estimated risk differences for obesity-related, colorectal and pancreatic, and kidney cancer were similar across sexes. Estimated risk difference for obesity-related cancers under the > 15 MET-hrs/wk strategy compared to no intervention was slightly greater in women than in men (Additional file 3: Table S6).

### Sensitivity analyses and positive control outcome

Estimates remained consistent across multiple sensitivity analyses (Additional file 3: Table S7). When evaluating CVD mortality as a positive outcome control, the estimated 11-year risk of CVD mortality was 5.8% under no intervention. Compared to no intervention, the estimated risk was higher by 0.99% (95% CI, 0.85% to 1.10%) under > 0– < 7.5 MET-hrs/wk strategy and lower by 0.59% (95% CI, − 0.78% to − 0.50%) and 1.88% (95% CI, − 2.14% to − 1.67%) under 7.5–15 MET-hrs/wk and > 15 MET-hrs/wk strategies, respectively (Additional file 3: Table S8). Estimates also remained similar after additional adjustments for parity and age at first birth in the analyses of breast and endometrial cancer outcomes (Additional file 3: Table S9).

## Discussion

In this large prospective cohort study emulating a target trial, we evaluated the long-term observational analog of a per-protocol effect of sustained MVPA strategies on the risk of obesity-related cancers. At both pre-intervention and baseline, more than half of eligible participants reported MVPA volumes meeting or exceeding physical activity recommendations for cancer prevention, and many would have maintained their MVPA in this range in the absence of intervention. We estimated that, compared to no intervention, consistently not meeting MVPA recommendations over 11 years may modestly increase the risk of all obesity-related cancers combined, particularly colorectal and breast cancers, but not endometrial, pancreatic, or kidney cancers. This effect was particularly apparent among participants who met the MVPA recommendations before the interventions. In contrast, compared to no intervention, meeting or exceeding the recommended MVPA volume did not affect obesity-related cancer incidence in the overall population; however, among individuals who had already met the recommendations prior to the intervention, it suggested a modest risk reduction for all obesity-related cancers combined and colorectal cancer, though not for other outcomes of interest. Analyses using “not meeting the recommendations” as the reference group revealed a more pronounced benefit of both meeting and exceeding the recommendations for these outcomes, likely reflecting the high proportion of participants who met the recommendations without intervention.

Obesity promotes cancer through a complex interplay of biological disruptions, including elevated insulin levels and insulin-like growth factors (IGF), chronic inflammation, and metabolic dysfunction [49]. Emerging evidence suggests that expanding adipose tissue fosters a tumor-supportive environment via enhanced tumor-promoting signaling [50] and hypoxia [49]. Regular physical activity has been shown to reduce circulating insulin, improve insulin sensitivity, lower IGF-1 bioavailability, and decrease inflammation; it may also enhance tissue oxygenation and vascular function, potentially offsetting adipose hypoxia [1]. Together these mechanisms support the plausibility of sustained MVPA as a modifiable strategy for preventing cancers mediated by excess adiposity [1].

To our knowledge, this is the first study to evaluate the long-term effects of sustained leisure-time MVPA intervention on obesity-related cancer as a combined outcome using an emulated target trial design. Prior large observational studies have reported strong inverse associations between higher baseline MVPA and risk of several site-specific cancers, including colon, rectum, breast, endometrium, and bladder cancers, but not pancreatic cancer [19, 51–53]. Our focus on all obesity-related cancers combined may partly explain the modest overall estimates observed, as this composite outcome includes both cancers with established inverse relationships with MVPA (e.g., colon, breast, endometrial) and others with more limited or inconsistent evidence (e.g., thyroid, rectal, pancreatic, meningeal, and ovarian cancers) [1].

To capture the outcomes likely driven by excess adiposity, we stratified our analyses by baseline BMI [43]. Among participants with overweight or obesity, compared to no intervention, sustaining MVPA below the recommended volume showed a modest increase in risk of all obesity-related cancers combined and breast cancer, supporting the potential role of MVPA for mitigating cancer risk mediated by adiposity. However, we estimated that meeting or exceeding MVPA recommendations did not influence obesity-related cancer incidence in this subgroup, possibly reflecting misclassification due to overreporting of vigorous activity among individuals with higher BMI [54].

Participants who were active before baseline appeared more likely to benefit from sustained MVPA meeting or exceeding recommendations, potentially due to cumulative physiological advantages such as lower chronic inflammation [55], enhanced glucose-insulin dynamics [56, 57], and other favorable hormonal regulations [58]. In contrast, individuals who were inactive at pre-baseline may require a longer intervention duration or higher MVPA volume to achieve similar benefits. Alternatively, overestimation of self-reported MVPA by previously inactive individuals [59] may have resulted in differential misclassification and attenuated estimated effects in this group.

While our MVPA strategies thresholds were based on current recommendations for cancer prevention, reflecting a range of minimum MVPA volume for the general population [10, 16], it is possible that greater volumes may be required to prevent specific cancers in this cohort. For example, an earlier CPS-II Nutrition Cohort study found a lower risk of postmenopausal breast cancer only among women reporting very high MVPA volumes (> 42 MET-hrs/wk vs. > 0–7.0 MET-hrs/wk), underscoring the challenge of sustaining such high MVPA volumes over time in this population [52]. Future studies may benefit from evaluating the effect of exceeding current recommendations at multiple thresholds.

Estimates from previous observational studies are not directly comparable to differences in design and analytic approach. While many studies report hazard ratios for the association of baseline MVPA and cancer outcomes, which may not be easy to interpret [60], our emulated target trial estimated absolute risk under hypothetical MVPA intervention sustained over 11 years of follow-up. To mirror the feasibility of interventions in a pragmatic trial, we considered dynamic strategies that varied according to individuals’ CVD status (rather than fixed strategies). In the presence of competing events, unlike earlier studies [19, 51, 52], we estimated total effects [40, 61], which account for all causal pathways, including those mediated through competing events. This approach may explain our modest effect estimates compared with studies that censor individuals once they experience a competing event [40, 61]. Notably, the 11-year absolute risk of postmenopausal breast cancer under no intervention (5.8%) was comparable to the 5.0% risk observed in the placebo group of the Women’s Health Initiative hormone therapy trial [62], suggesting our modeling approach produced reasonable estimates.

### Strengths

Additional strengths of our study include the use of rich longitudinal data from the CPS-II Nutrition Cohort with over 11 years of follow-up and 4344 verified obesity-related cancer diagnoses. The parametric g-formula allowed adjustment for time-varying confounding, appropriately handling treatment-confounder feedback from variables such as BMI, which may be affected by prior MVPA and may influence future MVPA [22]. Sensitivity analyses suggested the robustness of primary findings to scenarios involving new COPD diagnosis, age-related changes in MVPA, and adjustments for healthcare-seeking behavior. Emulated target trial design allowed exploration of the full spectrum of MVPA exposure, including lower-than-recommended MVPA volume that would be unethical to assign in a randomized trial. The strong protective link between MVPA and CVD mortality, used as a positive control outcome, further supported the internal validity of our estimates.

### Limitations

First, while target trial emulation provides a structured framework to clarify causal questions and reduce design-related biases, it does not eliminate biases inherent to the underlying observational data and is not equivalent to a randomized trial. Like any observational study, it relies on strong assumptions of exchangeability, positivity, consistency, and correct model specification, which may not always hold in practice [63]. We attempted to satisfy exchangeability between intervention groups at each follow-up through careful confounder adjustment, though residual confounding remains possible [64]. Second, MVPA was self-reported using a questionnaire that was not validated in the CPS-II Nutrition Cohort [28], which may introduce measurement error and misclassification [65]. Repeated biannual assessments may have mitigated the temporary reporting errors and recall bias. Third, model misspecification is possible, though our g-formula estimates under no intervention closely tracked the inverse probability weighted (IP-weighted) ones, and sensitivity analyses using different functional forms for covariates did not materially change estimates (Additional file 2: Fig. S5). Fourth, statistical power was not sufficient to estimate the risk of other site-specific obesity-related cancers (e.g., esophageal, gallbladder, liver, ovarian, gastric cardia, thyroid, meningioma, and multiple myeloma). Additionally, the number of endometrial, pancreatic, and kidney cancer cases was too small to estimate risk among participants with normal body weight. Fifth, follow-up was restricted to 11 years to emulate sustained adherence under the assumption that participants maintained their MVPA within the reported range for approximately 2 years after each assessment; this was feasible only during the period with consistent MVPA data collection between 1999 and 2011. Sixth, due to the lack of routinely collected waist circumference or waist-to-hip ratio data, BMI was used to assess excess adiposity, which may have introduced (likely non-differential) misclassification. Lastly, the cohort was predominantly older, white, and with higher educational attainment and MVPA, so our estimates may not be generalizable to other populations with different distributions of leisure-time MVPA or potential confounders.

## Conclusions

In this emulated target trial, we estimated that compared to the usual MVPA in the CPS-II Nutrition Cohort, engaging in less than 150 min of moderate-intensity or less than 75 min of vigorous-intensity activity per week over 11 years may increase the risk of all obesity-related cancers combined, as well as colorectal and breast cancer. In contrast, compared to not meeting the recommended activity volume, sustaining 150–300 min of moderate-intensity activity or 75–150 min of vigorous-intensity activity or higher per week over 11 years may help modestly reduce the risk of obesity-related cancers overall and colorectal cancer, particularly among older adults who were already physically active prior to the intervention period. Compared to maintaining usual activity, exceeding guideline recommendations may modestly reduce the 11-year risk of obesity-related cancers among older adults who were meeting or exceeding recommendations before the intervention. These results highlight the value of sustained adherence to physical activity guidelines and suggest that earlier initiation may enhance cancer prevention benefits. Future studies should examine the effect of interventions with longer follow-up in more diverse populations, incorporate objective measures of physical activity, and evaluate combined lifestyle interventions.

## Supplementary Information

Additional file 1: Additional methods. Sample program

Additional file 2: Fig. S1 Flowchart of the eligible women. Fig. S2 Simplified DAG. Fig. S3 Standardized event-free survival curves for all outcomes and physical activity intervention strategies. Fig. S4 Mean BMI and 95% CI over the follow-up and by baseline BMI categories. Fig. S5 Comparison between inverse probability weighted estimates and g-formula estimates under no intervention

Additional file 3: Table S1 Variables used to model the 11-year cancer outcomes. Table S2 Total number of person-years, events, losses to follow-up, competing events, and administrative censorings during the follow-up. Table S3 Sensitivity analysis using “not meeting the recommended MVPA volume” intervention strategy as a reference group. Table S4 Stratified analysis by pre-baseline MVPA using “not meeting the recommended MVPA volume” strategy as a reference group. Table S5 Stratified analysis by baseline BMI using “not meeting the recommended MVPA volume” strategy as a reference group. Table S6 Stratified analysis by sex. Table S7 Sensitivity analysis using alternative model specifications. Table S8 Sensitivity analysis using CVD mortality as a positive outcome control. Table S9 Sensitivity analysis further adjusting for parity and age at first birth

## Data Availability

Data are available from the American Cancer Society by following the ACS Data Access Procedures (https://www.cancer.org/content/dam/cancer-org/research/epidemiology/cancer-prevention-study-data-access-policies.pdf) for researchers who meet the criteria for access to confidential data. Please email cohort.data@cancer.org to inquire about access.

## Abbreviations

ACS: American Cancer Society
BMI: Body mass index
CPS-II: Cancer Prevention Study-II
CVD: Cardiovascular disease
IARC: International Agency for Research on Cancer
IQR: Interquartile range
MET: Metabolic equivalent of task
MET-hrs/wk: Metabolic equivalent of task hours per week
MVPA: Moderate-to-vigorous intensity physical activity
RCT: Randomized controlled trial
RR: Risk ratio
SDG: Sustainable development goal
WCRF: World Cancer Research Fund
