# Supplementary material for: Effectiveness of sustained leisure-time physical activity strategies for obesity-related cancer prevention: an emulated target trial in a prospective US cohort
Source: BMC Med. 2025 Oct 27;23:580. doi: 10.1186/s12916-025-04417-z (PMC12557993; doi:10.1186/s12916-025-04417-z)
Supplement: Supplementary file 1 — Additional file 1: Additional methods. Sample program [file 12916_2025_4417_MOESM1_ESM.docx]

**Effectiveness of sustained leisure-time physical activity strategies for obesity-related cancer prevention: an emulated target trial in a prospective US cohort**

Valeria Elahy, PhD; Yu-Han Chiu, MD, ScD; Alpa V. Patel, PhD; Erika Rees-Punia, PhD; Marjorie L. McCullough, ScD; Anita R. Peoples, PhD; Ying Wang, PhD

Table of Contents

[Supplementary Methods 2](#_Toc208315440)

[Sample Program (SAS) 3](#_Toc208315441)

[Supplementary References 6](#_Toc208315442)

**Additional Methods**

**Covariate Data Assessment**

BMI was calculated using self-reported height from the 1982 baseline survey and weight from the follow-up surveys. Smoking status was derived from the 2001 survey responses and categorized into 5 groups (never-smokers, former smokers quitting <10 years, 10-<20 years, ≥20 years, or current smokers). Diabetes status was identified by self-reported diabetes or self-reported use of insulin or oral diabetes medications, and CVD status was determined through a self-reported history of coronary artery disease, stroke, myocardial infarction, or transient ischemic attack. Missing data on diabetes and CVD were carried forward if previously reported. Diet quality was assessed in 1999 using the ACS guideline diet score (out of 12, with higher score indicating higher diet quality) originally developed by McCullough et al. to assess concordance with the ACS Nutrition and Physical Activity Guidelines for Cancer Prevention. [1] ACS guideline diet score was calculated using dietary data assessed using the FFQ in 1999 [2]. Concordance with the recommendation to follow a healthy eating pattern was assessed based on the intake of fruits and vegetables, whole grains, red and processed meats, sugar-sweetened beverages, highly processed foods, and refined grains.

**Sample Program (SAS)**

/*Define PA Interventions for primary analysis*/
/* Define Macro for Low Physical Activity Intervention if No CVD */
%macro lowPAnoCVD;
 /* Lower Bound Intervention */
 if (metrex=0) and (rand('uniform') <= 1) and (cvdn ne 1) then do;
 metrex = 1.0;
 smetrex[time] = metrex;
 metrex_totinterv = metrex_totinterv + 1;
 intervened = 1;
 intervenedk[time] = 1;
 totinterv = totinterv + 1;
 end;

if (metrex >= 7.5) and (rand('uniform') <= 1) and (cvdn ne 1) then do;
 metrex = 7;
 smetrex[time] = metrex;
 metrex_totinterv = metrex_totinterv + 1;
 intervened = 1;
 intervenedk[time] = 1;
 totinterv = totinterv + 1;
 end;
%mend;

%let interv1 = intno=1, nintvar=1,
 intlabel='PA >0-<7.5 Met-hrs/wk if no CVD',
 intvar1=metrex, inttype1=-1, inttimes1=0 1 2 3 4 5,
 intusermacro1=lowPAnoCVD;

/* Define Macro for Medium Physical Activity Intervention if No CVD */
%macro medPAnoCVD;
 /* Upper Bound Intervention */
 if (metrex > 15.00) and (rand('uniform') <= 1) and (cvdn ne 1) then do;
 metrex = 15.00;
 smetrex[time] = metrex;
 metrex_totinterv = metrex_totinterv + 1;
 intervened = 1;
 intervenedk[time] = 1;
 totinterv = totinterv + 1;
 end;

 /* Lower Bound Intervention */
 if (metrex < 7.5) and (rand('uniform') <= 1) and (cvdn ne 1) then do;
 metrex = 7.5;
 smetrex[time] = metrex;
 metrex_totinterv = metrex_totinterv + 1;
 intervened = 1;
 intervenedk[time] = 1;
 totinterv = totinterv + 1;
 end;
%mend;

%let interv2 = intno=2, nintvar=1,
 intlabel='PA 7.5-15 Met-hrs/wk if no CVD',
 intvar1=metrex, inttype1=-1, inttimes1=0 1 2 3 4 5,
 intusermacro1=medPAnoCVD;

/* Define Macro for High Physical Activity Intervention if No CVD */
%macro highPAnoCVD;
 if (metrex <= 15) and (rand('uniform') <= 1) and (cvdn ne 1) then do;
 metrex = 16;
 smetrex[time] = metrex;
 metrex_totinterv = metrex_totinterv + 1;
 intervened = 1;
 intervenedk[time] = 1;
 totinterv = totinterv + 1;
 end;
%mend;

%let interv3 = intno=3, nintvar=1,
 intlabel='PA >15 Met-hrs/wk if no CVD',
 intvar1=metrex, inttype1=-1, inttimes1=0 1 2 3 4 5,
 intusermacro1=highPAnoCVD;

/* Define Macro for Primary Analysis */
%macro primary (effect=, site=, effectname=, strataname=, sitenum=, strata=, extra=,
 bmiknots=, metrexknots=, alcspdknots=);

 /* G-Formula Call */
 %gformula(
 data=sample&site.&effectname.&strataname, /* Input dataset */
 id=id,
 time=time,
 timepoints=6,
 outc=event,
 outctype=binsurv,
 compevent=COMPD,
 compevent_cens=&effect, /* 1: Controlled Direct Effect, 0: Total Effect */
 censor=censor&site,
 refint=0,
 fixedcov=agegrp FAMCA_b GENDER educ RACEGRP bmicat_b metrex3cat_b2 alcspd_qb2 ACSDIET_qb2 dbn_b,
 timeptype=concat,
 ncov=5,
 cov1=metrex, cov1otype=4, cov1ptype=skpspl, cov1knots=&metrexknots, cov1skip=1 3,
 cov2=alcspd, cov2otype=4, cov2ptype=skpspl, cov2knots=&alcspdknots, cov2skip=0 2 4 5 ,
 cov3=cvdn, cov3otype=2, cov3ptype=tsswitch1,
 cov4=bmi, cov4otype=3, cov4ptype=lag1spl, cov4knots=&bmiknots,
 cov5=dbn, cov5otype=2, cov5ptype=tsswitch1,
 seed=1901,
 nsimul=,
 check_cov_models=0,
 print_cov_means=0,
 save_raw_covmean=0,
 resultsdata=ind&site.&strataname.&sysdate9,
 observed_surv=,
 betadata=,
 savelib=work,
 INTERVNAME=try,
 sample_start=0,
 sample_end=-1,
 numint=3,
 rungraphs=0,
 graphfile=ind&site.&effectname.&strataname.&sysdate9..pdf
 );

%mend primary;

/* OBESITY-RELATED CANCER */

/* Call Primary Analysis for Total Effect */

%primary (

effect=0,

extra=smkgrp_b,

bmiknots=20.81 25.18 31.60,

metrexknots=3.5 14.0 38.5,

alcspdknots=0.04 0.50 1.89,

SITE=OBES,

sitenum=2,

effectname=total,

strataname=all,

strata=0

);

**Supplementary References**

1. McCullough ML, Patel AV, Kushi LH, Patel R, Willett WC, Doyle C, et al. Association of socioeconomic and geographic factors with diet quality in US adults. JAMA Netw Open. 2022;5(6):e2216406.
2. Feskanich D, Rimm EB, Giovannucci EL, Colditz GA, Stampfer MJ, Litin LB, et al. Reproducibility and validity of food intake measurements from a semiquantitative food frequency questionnaire. J Am Diet Assoc. 1993;93(7):790-6.
