# Supplementary material for: Effectiveness of sustained leisure-time physical activity strategies for obesity-related cancer prevention: an emulated target trial in a prospective US cohort
Source: BMC Med. 2025 Oct 27;23:580. doi: 10.1186/s12916-025-04417-z (PMC12557993; doi:10.1186/s12916-025-04417-z)
Supplement: Supplementary file 2 — Additional file 2: Fig. S1 Flowchart of the eligible women. Fig. S2 Simplified DAG. Fig. S3 Standardized event-free survival curves for all outcomes and physical activity intervention strategies. Fig. S4 Mean BMI and 95% CI over the follow-up and by baseline BMI categories. Fig. S5 Comparison between inverse probability weighted estimates and g-formula estimates under no intervention [file 12916_2025_4417_MOESM2_ESM.docx]

**Effectiveness of sustained leisure-time physical activity strategies for obesity-related cancer prevention: an emulated target trial in a prospective US cohort**

Valeria Elahy, PhD; Yu-Han Chiu, MD, ScD; Alpa V. Patel, PhD; Erika Rees-Punia, PhD; Marjorie L. McCullough, ScD; Anita R. Peoples, PhD; Ying Wang, PhD

Table of Contents

[Fig. S1. Flowchart of the eligible women for the emulated target trial of recreational physical activity and breast or endometrial outcomes in the Cancer Prevention Study-II Nutrition Cohort (2001-2013) 2](#_Toc208316245)

[Fig. S2. Simplified causal directed acyclic graph encoding the assumed relationships of the effect of treatment A on the outcome of interest Y mediated by the competing events D. 3](#_Toc208316246)

[Fig. S3. Standardized event-free survival curves for all obesity-related cancers (a), colorectal (b), post-menopausal breast (c), endometrial (d), pancreatic (e) and kidney (f) cancer and physical activity intervention strategies in Cancer Prevention Study-II (2001-2013). 4](#_Toc208316247)

[Fig S4. Mean body mass index (BMI) and 95% confidence intervals over the follow-up overall and by baseline body mass index category in Cancer Prevention Study-II Nutrition Cohort (2001-2012). 5](#_Toc208316248)

[Fig. S5. Comparisons of risk of obesity-related cancers (a), competing events (death due to any cause other than obesity-related cancer) (b) and means of time-varying variables (c-g) between IP-weighted estimates and g-formula estimates under no intervention. 6](#_Toc208316249)

Women completed CPS-II Nutrition Cohort 1999 and 2001 surveys (n=77679)

BMI <18.5 in 2001 (n=1305)

Missing data on physical activity (n=2212) or other covariates (n=9211) in 1999 or 2001

BMI ≥18.5 kg/m^2^ in 2001 (n=50894)

Eligible for final analytic sample for breast (n= 38131) or endometrial (n= 38126) cancer outcomes

Cancer diagnosis (n=16043) or cardiovascular disease (heart attack, angina, coronary artery disease diagnosis, coronary bypass, angioplasty, stroke, or transient ischemic attack) (n=9437) before or in 2001

Eligible for physical activity intervention (n=52199)

BMI ≥18.5 kg/m^2^ in 2001 (n=39471)

No follow-up time after 2001 survey return for breast (n=1340) or endometrial (n=1343) cancer outcomes cohorts

Fig. S1. Flowchart of the eligible women for the emulated target trial of recreational physical activity and breast or endometrial outcomes in the Cancer Prevention Study-II Nutrition Cohort (2001-2013)

**A_k_**

**Y_k+1_**

**U**

**L_k_**

**D_k+1_**

**C_k+1_**

Fig. S2. Simplified causal directed acyclic graph encoding the assumed relationships of the effect of treatment A on the outcome of interest Y mediated by the competing events D. L_k:_ time-varying covariates at time k; A_k_: time-varying exposure (treatment) at time k; C_k+1:_ censoring at time k+1; D_k+1_: competing event at time k+1; Y^k+1^: outcome of interest at time k+1; U: unmeasured risk factors for time-varying covariates L and outcome Y.

Fig. S3. Standardized event-free survival curves for all obesity-related cancers (a), colorectal (b), post-menopausal breast (c), endometrial (d), pancreatic (e) and kidney (f) cancer and physical activity intervention strategies in Cancer Prevention Study-II (2001-2013). Estimates are based on the parametric g-formula adjusting for baseline (age, family history of cancer, sex, race, education, BMI, diabetes, smoking history) and pre-baseline (physical activity, diet quality, alcohol consumption) and time-varying covariates (BMI, physical activity, alcohol consumption, diabetes, CVD). The curves represent the probability of remaining cancer-free over time rather than time to mortality.

a.

b.

c.

d.

e.

f.


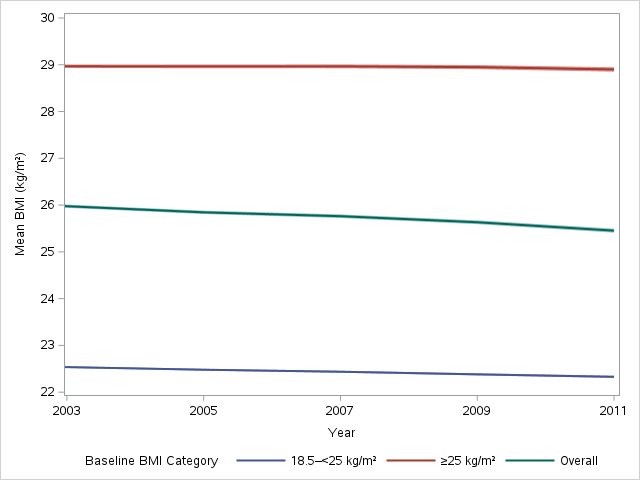


Fig S4. Mean body mass index (BMI) and 95% confidence intervals over the follow-up overall and by baseline body mass index category in Cancer Prevention Study-II Nutrition Cohort (2001-2012).


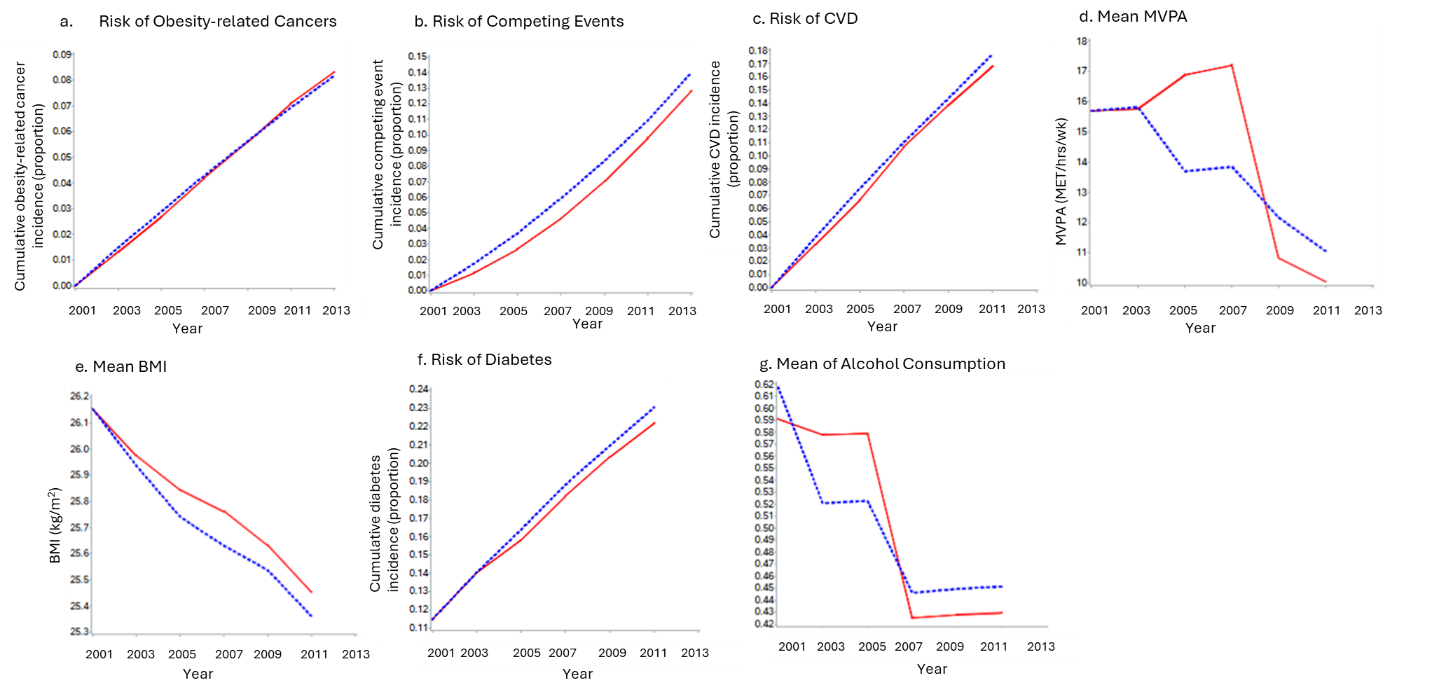


Fig. S5. Comparisons of risk of obesity-related cancers (a), competing events (death due to any cause other than obesity-related cancer) (b) and means of time-varying variables (c-g) between IP-weighted estimates and g-formula estimates under no intervention.
