# Supplementary material for: Effectiveness of sustained leisure-time physical activity strategies for obesity-related cancer prevention: an emulated target trial in a prospective US cohort
Source: BMC Med. 2025 Oct 27;23:580. doi: 10.1186/s12916-025-04417-z (PMC12557993; doi:10.1186/s12916-025-04417-z)
Supplement: Supplementary file 3 — Additional file 3: Table S1 Variables used to model the 11-year cancer outcomes. Table S2 Total number of person-years, events, losses to follow-up, competing events, and administrative censorings during the follow-up. Table S3 Sensitivity analysis using “not meeting the recommended MVPA volume” intervention strategy as a reference group. Table S4 Stratified analysis by pre-baseline MVPA using “not meeting the recommended MVPA volume” strategy as a reference group. Table S5 Stratified analysis by baseline BMI using “not meeting the recommended MVPA volume” strategy as a reference group. Table S6 Stratified analysis by sex. Table S7 Sensitivity analysis using alternative model specifications. Table S8 Sensitivity analysis using CVD mortality as a positive outcome control. Table S9 Sensitivity analysis further adjusting for parity and age at first birth [file 12916_2025_4417_MOESM3_ESM.docx]

**Effectiveness of sustained leisure-time physical activity strategies for obesity-related cancer prevention: an emulated target trial in a prospective US cohort**

Valeria Elahy, PhD; Yu-Han Chiu, MD, ScD; Alpa V. Patel, PhD; Erika Rees-Punia, PhD; Marjorie L. McCullough, ScD; Anita R. Peoples, PhD; Ying Wang, PhD

Table of Contents

[Table S1. Variables used to model the 11-year cancer outcomes, Cancer Prevention Study-II Nutrition Cohort (2001-2013). 2](#_Toc208316071)

[Table S3. Sensitivity analysis using “not meeting the recommended MVPA volume” (>0-<7.5MET-hrs/wk) intervention strategy as a reference group. 6](#_Toc208316072)

[Table S4. Stratified analysis by pre-baseline (1999) MVPA using “not meeting the recommended MVPA volume” (>0-<7.5 MET-hrs/wk) strategy as a reference group. 7](#_Toc208316073)

[Table S5. Stratified analysis by baseline (2001) BMI using “not meeting the recommended MVPA volume” (>0-<7.5MET-hrs/wk) strategy as a reference group. 8](#_Toc208316074)

[Table S6. Stratified analysis by sex. 9](#_Toc208316075)

[Table S7. Sensitivity analysis: estimated 11-year risks^1^ for all obesity-related cancers^2^ under leisure-time aerobic moderate-to-vigorous intensity physical activity (MVPA) strategies in the Cancer Prevention Study-II Nutrition Cohort (2001–2013). 10](#_Toc208316076)

[Table S8. Sensitivity analysis using cardiovascular disease (CVD) mortality as a positive outcome control. 12](#_Toc208316077)

[Table S9. Sensitivity analysis further adjusting for parity and age at first birth. 13](#_Toc208316078)

Table S1. Variables used to model the 11-year cancer outcomes, Cancer Prevention Study-II Nutrition Cohort (2001-2013).

| **Variable name** | **Years assessed** | **As dependent** | **As independent** |
| --- | --- | --- | --- |
| **Time-fixed (baseline)** | | | |
| Age | 2001 | Not predicted | 5 categories:  1=”<60”  2=”60-<65”  3=”65-<70”  4=”70-<75”  5=”≥75” |
| Sex | 1982 | Not predicted | 2 categories:  0=”Male”, 1=”Female” |
| Race | 1982 | Not predicted | 3 categories:  1=”White/White Hispanic”  2=”Black/Black Hispanic”  3=”Other/Unknown” |
| Education | 1982 | Not predicted | 3 categories:  1="HS Grad or lower "  2=”Some College”  3=”College Grad or unknown” |
| Family cancer history | 2001 | Not predicted | Indicator:  0=”No”, 1=”Yes” |
| Smoking history^8^ | 2001 | Not predicted | 6 categories:  1 = “Non-smoker”  2 = “Former smoker (quit <20 y ago and missing for quit time)”  3 = “Former smoker (quit 20-<30 y ago)”  4 = “Former smoker (quit ≥30 y ago)”  5 = “Current smoker (has smoked < 40 y or missing duration)”  6 = “Current smoker (has smoked ≥40 y)” |
| Smoking history | 2001 | Not predicted | 3 categories:  1 = “Non-smoker”  2 = “Former smoker  3 = “Current smoker” |
| BMI | 2001 | Not predicted | 3 categories:  0=" <25 kg/m^2^"  1=" 25-<30 kg/m^2^"  2=" >30 kg/m^2^" |
| Diabetes | 2001 | Not predicted | Indicator:  0=”No”, 1=”Yes” |
| **Time-fixed (pre-baseline)** | | | |
|  |  |  |  |
| Physical activity | 1999 | Not predicted | 3 categories:  0="0-< 7.5 MET-hrs/wk"  1=”7.5-15 MET-hrs/wk”  2=”>15 MET-hrs/wk” |
| Alcohol Consumption | 1999 | Not predicted | 3 percentile-based categories:  0=”0 servings/wk”  1=”0.07-<0.48 servings/wk”  2=”0.48-4.21 servings/wk” |
| ACS diet score^6^ | 1999 | Not predicted | 3 percentile-based categories:  0=”<=5.25”  1=”>5.25-7”  2=”>7” |
|  |  |  |  |
| Cancer screening^7^ | 1999 | Not predicted | Indicator:  0=”No”, 1=”Yes” |
| Parity^10^ | 1982 | Not predicted | 0=”Nulliparous”  2=”1-2 births”  3=”3 or more births”  4= “Missing” |
| Age at first birth^10^ | 1982 | Not predicted | 0=“Nulliparous”  1=”<20 years”  2=”20-24 years”  3=”25-29 years”  4=”30 or more years”  5=”Missing” |
| **Time-varying^2^** | | | |
| Physical activity | 2001-2011 (every 2y, excl 2003, 2007) | Logistic then log-linear^4^ | Restricted cubic spline, with knots at 1.75, 12.87 and 42.5 MET-hrs/wk |
| CVD | 1999-2011 (every 2y) | Logistic to failure^5^ | Indicator:  0=”No”, 1=”Yes”, + Product term between the indicator for diagnosis and time since diagnosis |
| BMI^5^ | 2001-2011 (every 2y) | Linear^3^ | Restricted cubic spline, with knots at 19.84, 25.37 and 34.18 kg/m^2^ |
| Diabetes | 2001-2011 (every 2y) | Logistic to failure^5^ | Indicator:  0=”No”, 1=”Yes”, + Product term between the indicator for diagnosis and time since diagnosis |
| Alcohol Consumption | 1999, 2003, 2007, 2013 | Logistic then log-linear | Restricted cubic spline, with knots at 0.07, 0.56 and 3.14 servings/day |
| Age^1^ | 2001-2011 (every 2y) | Linear^3^ | Restricted cubic spline, with knots at 64, 74 and 84 years |
| Cancer screening^7^ | 2001-2011 (every 2y) | Logistic | Indicator:  0=”No”, 1=”Yes” |

^1^ Only used for the estimates of the sensitivity analysis to estimate the effect of treatment only among the participants aged <90 years (Table S4).

^2^ When used as independent variables, values in the current and the previous period were included in the models. In the primary analysis, when modeling the joint distribution of time-varying covariates reported in the same questionnaire, we assumed the temporal ordering of covariates followed the order in this table. In sensitivity analyses, we altered the temporal ordering (Table S4).

^3^ Variables predicted by a linear model were assigned a value equal to the predicted value plus the standard error multiplied by a random number from a Normal (01) distribution. Therefore, two individuals with the same risk factor history were not necessarily predicted to have exactly the same risk factor value at the next time period.

Simulated values of continuous risk factors were truncated so that they did not fall outside of the observed range.

^4^ Variables with many zero values were predicted in two stages. First, we fitted a logistic model on an indicator that the variable is nonzero. Second, we fitted a linear model for the positive values. A log-transformed variable was created for use as the dependent variable in the linear model, and the resultant log-transformed predicted values were transformed back to the original scale. Simulated values were truncated so that they did not fall outside of the observed range.

^5^ BMI variable was modeled on the log scale in the sensitivity analysis (Table S4).

^6^ Out of 12.

^7^ Only used for the estimates of the sensitivity analysis further adjusting for cancer screening practices (Table S4).

^8^ Only used for the estimates of the sensitivity analysis excluding current and recent smokers further adjusting for the time since quit (Table S4).

^9^ Variables predicted by a logistic model were assigned a value of 1 if the predicted probability was greater than a random number from a uniform distribution. After the first 1 is generated, the value is set to 1 thereafter.

^10^ Only used for the estimates of the sensitivity analysis further adjusting for parity and age at first birth (Table S6).

Table S2. Total number of person-years, events, losses to follow-up, competing events, and administrative censorings during the 11-year follow-up among the participants eligible for the emulated target trial in Cancer Prevention Study-II Nutrition Cohort (2001-2013).

| **Outcome** | **Number of eligible individuals** | **Person‑years** | **Number of events** | **Number of censoring events^1^** | **Number of competing events^2^** | **Number of administrative censorings on June 30, 2013** |
| --- | --- | --- | --- | --- | --- | --- |
| Obesity-related cancer | 60958 | 558100.86 | 4344 | 17769 | 6431 | 32414 |
| Colorectal cancer | 60996 | 561908.62 | 876 | 20273 | 6953 | 32894 |
| Breast cancer | 38131 | 361372.50 | 1936 | 10822 | 3362 | 22011 |
| Endometrial cancer | 38126 | 363088.19 | 295 | 12216 | 3377 | 22238 |
| Pancreatic cancer | 61013 | 562854.86 | 320 | 20858 | 6849 | 32986 |
| Kidney cancer | 61006 | 562711.47 | 236 | 20758 | 7041 | 32971 |
| CVD mortality^3^ | 60897 | 597597.86 | 2791 | 14322 | 6204 | 37580 |

^1^ Censoring events are defined as non-response to a CPS-II Nutrition Cohort follow-up survey, report of non-verified cancer diagnosis, or report of cancer other than a cancer of interest.

^2^ Competing events are defined as a death due to any cause other than the outcome of interest.

^3^ Positive outcome control.

Table S3. Sensitivity analysis using “not meeting the recommended MVPA volume” (>0-<7.5MET-hrs/wk) intervention strategy as a reference group. Estimated 11-year risks^1^ of all obesity-related cancers, female postmenopausal breast cancer, colorectal cancer, pancreatic cancer, endometrial cancer, and kidney cancer under leisure-time aerobic moderate-to-vigorous intensity physical activity (MVPA) strategies compared to “not meeting the recommended MVPA volume” in the Cancer Prevention Study-II Nutrition Cohort (2001–2013).

| **Intervention** | **Risk Difference (95% CI)** |
| --- | --- |
| **Obesity-related Cancers** |  |
| 7.5–15 MET-hrs/wk vs. not meeting the recommended MVPA | -0.11 (-0.34, 0.05) |
| >15 MET-hrs/wk vs. not meeting the recommended MVPA | -0.36 (-0.79, -0.05) |
| **Colorectal Cancer** |  |
| 7.5–15 MET-hrs/wk vs. not meeting the recommended MVPA | -0.13 (-0.29, -0.01) |
| >15 MET-hrs/wk vs. not meeting the recommended MVPA | -0.26 (-0.50, -0.07) |
| **Breast Cancer** |  |
| 7.5–15 MET-hrs/wk vs. not meeting the recommended MVPA | -0.06 (-0.33, 0.13) |
| >15 MET-hrs/wk vs. not meeting the recommended MVPA | -0.26 (-0.63, 0.08) |
| **Endometrial Cancer** |  |
| 7.5–15 MET-hrs/wk vs. not meeting the recommended MVPA | 0.06 (-0.01, 0.19) |
| >15 MET-hrs/wk vs. not meeting the recommended MVPA | 0.10 (-0.01, 0.40) |
| **Pancreatic Cancer** |  |
| 7.5–15 MET-hrs/wk vs. not meeting the recommended MVPA | 0.04 (-0.01, 0.12) |
| >15 MET-hrs/wk vs. not meeting the recommended MVPA | 0.07 (-0.03, 0.18) |
| **Kidney Cancer** |  |
| 7.5–15 MET-hrs/wk vs. not meeting the recommended MVPA | 0.02 (-0.04, 0.08) |
| >15 MET-hrs/wk vs. not meeting the recommended MVPA | 0.01 (-0.08, 0.12) |

^1^ Estimates are based on the parametric g-formula adjusting for baseline (age, family history of cancer, sex, race, education, BMI, diabetes, and smoking history) and pre-baseline (physical activity, diet quality, alcohol consumption) and time-varying covariates (BMI, physical activity, alcohol consumption, diabetes, CVD).

Abbreviations: CI, confidence interval; MET, metabolic equivalent of task; MVPA, moderate-to-vigorous intensity physical activity.

Table S4. Stratified analysis by pre-baseline (1999) MVPA using “not meeting the recommended MVPA volume” (>0-<7.5 MET-hrs/wk) strategy as a reference group. Estimated^1^ 11-year risk differences for all obesity-related cancers, female postmenopausal breast cancer, colorectal cancer, pancreatic cancer, endometrial cancer, and kidney cancer under leisure-time aerobic moderate-to-vigorous intensity physical activity (MVPA) strategies compared to “not meeting the recommended MVPA volume” in the Cancer Prevention Study-II Nutrition Cohort (2001-2013).

|  | **Estimated 11-year Risk Difference (95% CI), %** | |
| --- | --- | --- |
| **Treatment Strategy** | **Pre-baseline MVPA <7.5 MET-hrs/wk** | **Pre-baseline MVPA ≥7.5 MET-hrs/wk** |
| **Obesity-Related Cancer** |  |  |
| 7.5-15 MET-hrs/wk vs. not meeting the recommended MVPA | -0.01 (-0.34, 0.42) | -0.20 (-0.49, -0.01) |
| >15 MET-hrs/wk vs. not meeting the recommended MVPA | -0.08 (-0.80, 0.54) | -0.55 (-1.07, -0.16) |
| **Colorectal cancer** |  |  |
| 7.5-15 MET-hrs/wk vs. not meeting the recommended MVPA | -0.07 (-0.25, 0.09) | -0.19 (-0.31, -0.07) |
| >15 MET-hrs/wk vs. not meeting the recommended MVPA | -0.13 (-0.39, 0.21) | -0.35 (-0.51, -0.15) |
| **Breast Cancer** |  |  |
| 7.5-15 MET-hrs/wk vs. not meeting the recommended MVPA | 0.18 (-0.15, 0.53) | -0.16 (-0.41, 0.32) |
| >15 MET-hrs/wk vs. not meeting the recommended MVPA | 0.01 (-0.71, 0.51) | -0.45 (-1.00, 0.31) |
| **Endometrial Cancer** |  |  |
| 7.5-15 MET-hrs/wk vs. not meeting the recommended MVPA | 0.10 (-0.12, 0.39) | 0.05 (-0.05, 0.17) |
| >15 MET-hrs/wk vs. not meeting the recommended MVPA | 0.02 (-0.34, 0.48) | 0.11 (-0.10, 0.33) |
| **Pancreatic Cancer** |  |  |
| 7.5-15 MET-hrs/wk vs. not meeting the recommended MVPA | -0.04 (-0.14, 0.10) | 0.09 (0.02, 0.16) |
| >15 MET-hrs/wk vs. not meeting the recommended MVPA | -0.05 (-0.28, 0.13) | 0.13 (0.00, 0.27) |
| **Kidney Cancer** |  |  |
| 7.5-15 MET-hrs/wk vs. not meeting the recommended MVPA | -0.01 (-0.10, 0.10) | 0.02 (-0.06, 0.08) |
| >15 MET-hrs/wk vs. not meeting the recommended MVPA | 0.04 (-0.09, 0.20) | -0.01 (-0.16, 0.09) |

^1^ Estimates are based on the parametric g-formula adjusting for baseline (age, family history of cancer, sex, race, education, BMI, diabetes, smoking history) and pre-baseline (physical activity, diet quality, alcohol consumption) and time-varying covariates (BMI, physical activity, alcohol consumption, diabetes, CVD). Individuals were not censored upon the development of a competing event to estimate the total effect of the intervention. Nonparametric bootstrapping was used to calculate 95% confidence intervals for all estimates.

Abbreviations: CI, confidence interval; MET, metabolic equivalent of task; MVPA, moderate-to-vigorous intensity physical activity.

Table S5. Stratified analysis by baseline (2001) BMI using “not meeting the recommended MVPA volume” (>0-<7.5MET-hrs/wk) strategy as a reference group. Estimated^1^ 11-year risk differences for all obesity-related cancers, female postmenopausal breast cancer, colorectal cancer, pancreatic cancer, endometrial cancer, and kidney cancer under leisure-time aerobic moderate-to-vigorous intensity physical activity (MVPA) strategies compared to “not meeting the recommended MVPA volume” in the Cancer Prevention Study-II Nutrition Cohort (2001-2013).

|  | **Estimated 11-year Risk Difference (95% CI), %** | |
| --- | --- | --- |
| **Treatment Strategy** | **Baseline BMI 18.5<25 kg/m^2^** | **Baseline BMI ≥25 kg/m^2^** |
| **Obesity-Related Cancer** |  |  |
| 7.5-15 MET-hrs/wk vs. not meeting the recommended MVPA | -0.08 (-0.33, 0.22) | -0.11 (-0.36, 0.22) |
| >15 MET-hrs/wk vs. not meeting the recommended MVPA | -0.21 (-0.72, 0.38) | -0.43 (-1.00, 0.07) |
| **Colorectal cancer** |  |  |
| 7.5-15 MET-hrs/wk vs. not meeting the recommended MVPA | -0.27 (-0.42, -0.07) | -0.01 (-0.12, 0.15) |
| >15 MET-hrs/wk vs. not meeting the recommended MVPA | -0.42 (-0.69, -0.08) | -0.12 (-0.29, 0.16) |
| **Breast Cancer** |  |  |
| 7.5-15 MET-hrs/wk vs. not meeting the recommended MVPA | 0.14 (-0.17, 0.41) | -0.26 (-0.58, 0.25) |
| >15 MET-hrs/wk vs. not meeting the recommended MVPA | 0.14 (-0.36, 0.66) | -0.61 (-1.09, 0.01) |
| **Endometrial Cancer^2^** |  |  |
| 7.5-15 MET-hrs/wk vs. not meeting the recommended MVPA | - | 0.04 (-0.13, 0.30) |
| >15 MET-hrs/wk vs. not meeting the recommended MVPA | - | 0.10 (-0.15, 0.49) |
| **Pancreatic Cancer^2^** |  |  |
| 7.5-15 MET-hrs/wk vs. not meeting the recommended MVPA | - | 0.08 (0.00, 0.18) |
| >15 MET-hrs/wk vs. not meeting the recommended MVPA | - | 0.11 (-0.06, 0.27) |
| **Kidney Cancer^2^** |  |  |
| 7.5-15 MET-hrs/wk vs. not meeting the recommended MVPA | - | 0.02 (-0.06, 0.12) |
| >15 MET-hrs/wk vs. not meeting the recommended MVPA | - | -0.01 (-0.13, 0.14) |

^1^ Estimates are based on the parametric g-formula adjusting for baseline (age, family history of cancer, sex, race, education, BMI, diabetes, smoking history) and pre-baseline (physical activity, diet quality, alcohol consumption) and time-varying covariates (BMI, physical activity, alcohol consumption, diabetes, CVD). Individuals were not censored upon the development of a competing event to estimate the total effect of the intervention. Nonparametric bootstrapping was used to calculate 95% confidence intervals for all estimates.

^2^ Estimates for endometrial, pancreatic, and kidney cancer among participants with BMI <25 kg/m² are not presented due to quasi-complete separation in the data, which indicates a violation of the positivity assumption. In these strata, sparse data limited the ability to support the modeled interventions without strong extrapolation, resulting in unstable and potentially unreliable estimates.

Abbreviations: CI, confidence interval; MET, metabolic equivalent of task; MVPA, moderate-to-vigorous intensity physical activity.

Table S6. Stratified analysis by sex. Estimated^1^ 11-year risk differences for all obesity-related cancers, female postmenopausal breast cancer, colorectal cancer, pancreatic cancer, endometrial cancer, and kidney cancer under leisure-time aerobic moderate-to-vigorous intensity physical activity (MVPA) compared to no intervention^2^ in the Cancer Prevention Study-II Nutrition Cohort (2001-2013).

|  | **Estimated 11-year Risk Difference (95% CI), %** | |
| --- | --- | --- |
| **Treatment Strategy** | **Men** | **Women** |
| **Obesity-Related Cancer^3^** |  |  |
| >0-<7.5 MET-hrs/wk vs. no intervention | 0.17 (-0.10, 0.40) | 0.20 (-0.02, 0.38) |
| 7.5-15 MET-hrs/wk vs. no intervention | 0.19 (0.01, 0.37) | -0.01 (-0.24, 0.21) |
| >15 MET-hrs/wk vs. no intervention | -0.01 (-0.27, 0.26) | -0.28 (-0.64, 0.13) |
| **Colorectal cancer^3^** |  |  |
| >0-<7.5 MET-hrs/wk vs. no intervention | 0.19 (0.02, 0.36) | 0.05 (-0.03, 0.14) |
| 7.5-15 MET-hrs/wk vs. no intervention | 0.07 (-0.05, 0.19) | -0.10 (-0.19, -0.01) |
| >15 MET-hrs/wk vs. no intervention | -0.13 (-0.33, 0.06) | -0.17 (-0.33, -0.02) |
| **Pancreatic Cancer^3^** |  |  |
| >0-<7.5 MET-hrs/wk vs. no intervention | -0.05 (-0.15, 0.04) | 0.00 (-0.06, 0.05) |
| 7.5-15 MET-hrs/wk vs. no intervention | 0.05 (-0.02, 0.13) | 0.01 (-0.05, 0.06) |
| >15 MET-hrs/wk vs. no intervention | 0.10 (-0.02, 0.23) | 0.01 (-0.10, 0.14) |
| **Kidney Cancer^3^** |  |  |
| >0-<7.5 MET-hrs/wk vs. no intervention | -0.04 (-0.13, 0.05) | 0.02 (-0.01, 0.05) |
| 7.5-15 MET-hrs/wk vs. no intervention | 0.05 (-0.01, 0.12) | 0.00 (-0.04, 0.05) |
| >15 MET-hrs/wk vs. no intervention | 0.09 (-0.03, 0.22) | -0.03 (-0.09, 0.05) |

^1^ Estimates are based on the parametric g-formula adjusting for baseline (age, family history of cancer, race, education, BMI, diabetes, smoking history) and pre-baseline (physical activity, diet quality, alcohol consumption) and time-varying covariates (BMI, physical activity, alcohol consumption, diabetes, CVD). Individuals were not censored upon the development of a competing event to estimate the total effect of the intervention. Nonparametric bootstrapping was used to calculate 95% confidence intervals for all estimates.

^2^ No intervention or "natural course" refers to observing and analyzing the effect of natural physical activity in the eligible population without any intervention.

^3^ Observed 11-year inverse probability weighted risks under the no intervention strategy and sample sizes were as follows: for obesity-related cancer, 4.5% in men (N=22,856) and 10.4% in women (N=38,102); for colorectal cancer, 1.9% in men (N=22,862) and 1.6% in women (N=38,134); for pancreatic cancer, 0.7% in men (N=22,872) and 0.6% in women (N=38,141); and for kidney cancer, 0.7% in men (N=22,868) and 0.3% in women (N=38,138).

Abbreviations: CI, confidence interval; MET, metabolic equivalent of task; MVPA, moderate-to-vigorous intensity physical activity.

Table S7. Sensitivity analysis: estimated 11-year risks^1^ for all obesity-related cancers^2^ under leisure-time aerobic moderate-to-vigorous intensity physical activity (MVPA) strategies in the Cancer Prevention Study-II Nutrition Cohort (2001–2013).

| **Sensitivity analyses type** | **Estimated 11-year Risk (%) (95% CI)** | | | | **Estimated 11-year Risk Difference (95% CI), %** | | | |
| --- | --- | --- | --- | --- | --- | --- | --- | --- |
|  | **No intervention** | **>0-<7.5 MET-hrs/wk** | **7.5-15 MET-hrs/wk** | **>15 MET-hrs/wk** | | **>0-<7.5 MET-hrs/wk vs. no intervention** | **7.5-15 MET-hrs/wk vs. no intervention** | **>15 MET-hrs/wk vs. no intervention** |
| 0. Primary | 8.2 (8.0, 8.4) | 8.4 (8.1, 8.7) | 8.3 (8.0, 8.5) | 8.0 (7.6, 8.3) | | 0.18 (0.05, 0.37) | 0.08 (-0.05, 0.19) | -0.18 (-0.44, 0.01) |
| 1. Minimum latency period analysis of 2 years^1^ | 7.1 (6.8, 7.3) | 7.1 (6.9, 7.4) | 7.1 (6.9, 7.4) | 7.0 (6.7, 7.3) | | 0.08 (-0.06, 0.25) | 0.04 (-0.07, 0.13) | -0.06 (-0.28, 0.15) |
| 2. Functional form^2^ | 8.2 (8.0, 8.4) | 8.4 (8.2, 8.8) | 8.3 (8.0, 8.6) | 8.1 (7.7, 8.4) | | 0.18 (0.05, 0.37) | 0.07 (-0.05, 0.20) | -0.17 (-0.44, 0.03) |
| 3. Order^3^ | 8.2 (7.9, 8.4) | 8.4 (8.1, 8.7) | 8.2 (7.9, 8.5) | 8.0 (7.6, 8.3) | | 0.18 (0.05, 0.36) | 0.07 (-0.05, 0.18) | -0.18 (-0.44, 0.01) |
| 4. Restrict to participants aged <90 years old at baseline (n=60942) and excuse them from adherence to the recommended physical activity level upon age 90 | 8.3 (8.0, 8.5) | 8.4 (8.1, 8.7) | 8.3 (8.1, 8.6) | 8.2 (7.8, 8.5) | | 0.16 (-0.03, 0.30) | 0.08 (-0.04, 0.20) | -0.12 (-0.36, 0.13) |
| 5. Excusing participants with COPD^4^ | 8.2 (7.9, 8.4) | 8.3 (8.1, 8.7) | 8.2 (7.9, 8.5) | 8.0 (7.6, 8.3) | | 0.19 (0.06, 0.37) | 0.07 (-0.05, 0.17) | -0.19 (-0.42, 0.00) |
| 6. Not excusing participants with CVD and including individuals with history of CVD in the analytic sample (n=81659) | 7.9 (7.7, 8.0) | 8.0 (7.8, 8.2) | 8.0 (7.7, 8.2) | 7.8 (7.4, 8.2) | | 0.13 (-0.01, 0.23) | 0.13 (-0.02, 0.27) | -0.06 (-0.31, 0.27) |
| 7. Further adjustment for cancer screening | 8.2 (7.9, 8.4) | 8.4 (8.2, 8.7) | 8.3 (8.0, 8.5) | 8.0 (7.7, 8.4) | | 0.17 (0.04, 0.36) | 0.07 (-0.04, 0.19) | -0.16 (-0.43, 0.01) |
| 8. Excluding current or recent smokers (quit <20y before baseline) at baseline | 8.2 (8.0, 8.4) | 8.4 (8.1, 8.7) | 8.3 (8.0, 8.6) | 8.0 (7.7, 8.4) | | 0.20 (0.01, 0.38) | 0.06 (-0.06, 0.19) | -0.22 (-0.41, 0.04) |

^1^ Treatment variables and other covariates except (history of cardiovascular events) were lagged by 2 years (one survey term). The g-formula risks and risk differences were therefore estimated over 9 years instead of over 11 years, as in the primary analysis.

^2^ Pre-baseline and time-varying BMI were modeled using the natural logarithm of BMI.

^3^ In the primary analysis, when modeling the joint distribution of time-varying covariates reported in the same questionnaire, we considered the temporal order that corresponds to the ordering of variables within Table S1. In sensitivity analyses, we reversed the temporal order of the time-varying intervention variables.

^4^ In the primary analysis, individuals were excused from following the physical activity intervention upon the development of a cardiovascular event (such as heart attack, angina, coronary artery disease diagnosis, coronary bypass, angioplasty, stroke, and transient ischemic attack). In sensitivity analysis, we required adherence to the assigned treatment strategies regardless of the CVD status.

Abbreviations: CPS-II, Cancer Prevention Study-II; MVPA, moderate-to-vigorous intensity physical activity.

Table S8. Sensitivity analysis using cardiovascular disease (CVD) mortality as a positive outcome control. Estimated 11-year risks^1^ of CVD mortality under leisure-time aerobic moderate-to-vigorous intensity physical activity (MVPA) strategies compared to no intervention in the Cancer Prevention Study-II Nutrition Cohort (2001–2013).

| **Intervention** | **Estimated risk (95% CI), %** | **Risk Ratio (95% CI), %** | **Risk Difference (95% CI)** | **Average % intervened on** | **Cumulative % intervened on^4^** |
| --- | --- | --- | --- | --- | --- |
| **CVD mortality** |  |  |  |  |  |
| No intervention^3^ | 5.8 (5.6, 6.0) | 1.00 (Reference) | 0 (Reference) | 0 | 0 |
| >0-<7.5MET-hrs/wk | 6.8 (6.5, 7.0) | 1.17 (1.15, 1.19) | 0.99 (0.85, 1.10) | 37.2 | 93.7 |
| 7.5-15 MET-hrs/wk | 5.2 (4.9, 5.4) | 0.90 (0.87, 0.91) | -0.59 (-0.78, -0.50) | 43.7 | 97.1 |
| >15 MET-hrs/wk | 3.9 (3.7, 4.1) | 0.67 (0.63, 0.71) | -1.88 (-2.14, -1.67) | 37.3 | 90.6 |

^1^ Estimates are based on the parametric g-formula adjusting for baseline (age, family history of cancer, sex, race, education, BMI, diabetes, smoking history) and pre-baseline (physical activity, diet quality, alcohol consumption) and time-varying covariates (BMI, physical activity, alcohol consumption, diabetes, CVD).

^2^ Among 60897 participants, there were 2791 CVD deaths during follow-up. The inverse probability weighted observed 11-year risk of CVD mortality was 5.3%.

^3^ No intervention is the observed physical activity in the population eligible for the target trial emulation.

^4^ Cumulative proportion of eligible participants who would have to change their physical activity level at any follow-up period to keep adhering to the specific intervention strategy.

Abbreviations: CI, confidence interval; CVD, cardiovascular disease; MET, metabolic equivalent of task; MVPA, moderate-to-vigorous intensity physical activity.

Table S9. Sensitivity analysis further adjusting for parity and age at first birth. Estimated 11-year risks^1^ of postmenopausal breast and endometrial cancers under leisure-time aerobic moderate-to-vigorous intensity physical activity (MVPA) strategies compared to no intervention^2^ in the Cancer Prevention Study-II Nutrition Cohort (2001–2013).

| **Intervention** | **Risk Difference (95% CI)** |
| --- | --- |
| **Breast Cancer** |  |
| >0-<7.5 MET-hrs/wk vs. no intervention^2^ | 0.14 (0.04, 0.28) |
| 7.5-15 MET-hrs/wk vs. no intervention^2^ | 0.08 (-0.16, 0.22) |
| >15 MET-hrs/wk vs. no intervention^2^ | -0.11 (-0.39, 0.13) |
| **Endometrial Cancer** |  |
| >0-<7.5 MET-hrs/wk vs. no intervention^2^ | -0.03 (-0.12, 0.01) |
| 7.5-15 MET-hrs/wk vs. no intervention^2^ | 0.03 (-0.05, 0.10) |
| >15 MET-hrs/wk vs. no intervention^2^ | 0.08 (-0.01, 0.28) |

^1^ Estimates are based on the parametric g-formula adjusting for baseline (age, family history of cancer, sex, race, education, BMI, diabetes, smoking history, parity and age at first birth) and pre-baseline (physical activity, diet quality, alcohol consumption) and time-varying covariates (BMI, physical activity, alcohol consumption, diabetes, CVD).

^2^ No intervention is the observed physical activity in the population eligible for the target trial emulation.

Abbreviations: CI, confidence interval; MET, metabolic equivalent of task; MVPA, moderate-to-vigorous intensity physical activity.
